# Hypoxia reconstructed colorectal tumor microenvironment weakening anti-tumor immunity: construction of a new prognosis predicting model through transcriptome analysis

**DOI:** 10.3389/fimmu.2024.1425687

**Published:** 2024-12-06

**Authors:** Ruizhi Zhang, Yisong Gao, Chong Li, Ruikang Tao, Gan Mao, Tianyu Song, Wenxiang Nie, Suao Liu, Kaixiong Tao, Wei Li

**Affiliations:** ^1^ Department of Gastrointestinal Surgery, Union Hospital, Tongji Medical College, Huazhong University of Science and Technology, Wuhan, China; ^2^ Center for Biomolecular Science and Engineering, University of California Santa Cruz, Santa Cruz, CA, United States

**Keywords:** hypoxia, colorectal cancer, tumor microenvironment immunity, extracellular matrix, WGCNA

## Abstract

**Background:**

Hypoxia in the tumor microenvironment (TME) plays a pivotal role in the progression and prognosis of colorectal cancer (CRC). However, effective methods for assessing TME hypoxia remain lacking. This study aims to develop a novel hypoxia-related prognostic score (HPS) based on hypoxia-associated genes to improve CRC prognostication and inform treatment strategies.

**Methods:**

Transcriptomic data from CRC patients were analyzed using Lasso regression to identify hypoxia-associated genes with the strongest prognostic significance. The identified genes were validated *in vitro* by assessing their expression under normoxic and hypoxic conditions in normal intestinal epithelial cells and CRC tumor cell lines. Functional relevance was explored through differential gene expression analysis, Gene Ontology (GO) and Kyoto Encyclopedia of Genes and Genomes (KEGG) enrichment analyses, and protein-protein interaction (PPI) network construction. The association of HPS with extracellular matrix (ECM) composition, immune cell infiltration, and immune suppression was also investigated.

**Results:**

Seven hypoxia-associated signature genes were identified, each demonstrating a strong correlation with CRC prognosis. The hypoxia-related prognostic score (HPS), derived from these genes, was significantly linked to changes in the TME. Specifically, HPS values were associated with alterations in ECM composition and distinct immune cell infiltration patterns. Higher HPS values corresponded to increased infiltration of immune-suppressive cells and reduced presence of anti-tumor immune cells. This imbalance promoted an immune-suppressive TME, facilitating tumor progression and immune evasion.

**Conclusions:**

The hypoxia-related prognostic score (HPS) captures the regulatory influence of TME hypoxia on immune responses, offering valuable insights into its role in tumor progression. HPS holds promise as a prognostic tool and a guide for developing personalized treatment strategies in CRC.

## 1 Introduction

Hypoxia, a critical component of the tumor microenvironment, is a result of imbalance between increased oxygen usage and insufficient oxygen supply drove by rapid and unlimited growth of tumor cells and lack of blood supply (1). This reciprocal interplay affects patient outcomes across various tumor types, significantly influencing tumor prognosis (2). Microenvironmental hypoxia is a factor that affects the prognosis of patients with various malignancies, including colorectal cancer (CRC) (3–5).

Hypoxia plays a pivotal role in driving tumor progression, orchestrating the growth and differentiation of tumor cells through various molecular mechanisms. Proliferation, invasion, and epithelial-mesenchymal transition of cancer cell are all associated with hypoxia and are closely linked to local tumor progression and distant metastasis (6–8). Moreover, hypoxia is involved in regulating different forms of tumor cell death, including apoptosis (9). Hypoxic cancer cells exhibit decreased levels of apoptosis and ferroptosis while autophagy levels increase, promoting their adaptation to the hypoxic TME (10–12).

Beyond its influence on cancer cells, hypoxia exerts significant effects on various other cells within the TME, including interstitial and immune cells. Hypoxia suppresses both the infiltration and functionality of immune cells, thereby critically influencing the tumor immune within the tumor microenvironment (13, 14). Furthermore, hypoxia can alter the matrix composition within the TME, leading to its remodeling (3).

Despite the critical role of TME hypoxia in tumor progression, detection techniques remain relatively inadequate (4). Surgical specimens are evaluated for hypoxia using immunohistochemistry or immunofluorescence to detect HIF1α expression (15). Although pimonidazole staining is utilized in animal experiments for hypoxia assessment, its clinical application remains limited (16). In our study, to enhance the assessment of hypoxia within the TME, obtain more precise tumor molecular classifications, and subsequently optimize the treatment of CRC patients, LASSO regression was employed. This method allowed us to screen for prognosis-associated genes, integrating clinicopathological characteristics to predict patient outcomes. Furthermore, we explored the mechanistic underpinnings of these genes through functional analysis.

## 2 Materials and methods

### 2.1 Data collection and preprocessing

Expression profiles of the GSE17536 and GSE14333 datasets were downloaded from the Gene Expression Omnibus database (GEO). The GEO dataset GSE17536 included 177 CRC samples, and the other GEO dataset GSE14333 included 290 CRC samples. TCGA-COAD and GTEx transcriptome cohort data were downloaded from the UCSC Xena website (https://xenabrowser.net/datapages/). The TCGA dataset included 616 CRC samples, and the GTEx dataset included 686 non-diseased colon tissue samples. All raw data were normalized and standardized using the R software packages including “limma” and “DESeq2”.

### 2.2 Single sample gene set enrichment analysis

The R package “GSVA” facilitated single-sample gene set enrichment analysis (ssGSEA) to investigate tumor-related pathway enrichment and immune cell infiltration within the GSE17536 dataset. We sourced tumor-related datasets from the hallmark gene sets in the MSigDB database [https://www.gsea-msigdb.org/gsea/msigdb].

### 2.3 Weighted gene co-expression networks analysis

The weighted gene co-expression network analysis (WGCNA) was constructed using the GSE17536 dataset. Among all the soft threshold values, we selected the β value with the highest mean connectivity (β = 13). The minimum number of genes was set at 30 to ensure the high reliability of the results. All genes were then divided into modules, each named by a different color. For further quantification of hypoxia-related genes and modules, only genes with a p-value of less than 0.001 were retained for subsequent analysis.

### 2.4 Establishment and validation of a colorectal cancer prognostic predictive signature

Univariate Cox regression analysis identified cancer hallmarks related to disease-specific survival (DSS) and overall survival (OS). We applied Lasso penalized Cox regression analysis to select hypoxia-related genes associated with prognosis. Subsequently, we used the LASSO Cox regression model to identify genes highly correlated with hypoxia and to construct the hypoxia-related prognosis score (HPS). We calculated the HPS score for each patient using the formula: HPS score =∑(coefficient × mRNA expression).

### 2.5 Construction of nomogram for colorectal cancer prognosis prediction

Hypoxia score and relevant clinical parameters were used to construct a nomogram, using the “survival” and the “rms” package of R. The nomogram was constructed to estimate 1-, 3-, and 5-year survival probabilities. The model’s performance was evaluated by using the calibration curve and C-index to assess the survival probabilities.

### 2.6 Gene set enrichment analysis

The function of hypoxia-related genes was explored using gene set enrichment analysis (GSEA). Differential gene expression profiles in the training and validation cohorts were analyzed using the R software package “clusterProfiler” (17). P-values < 0.05 and FDR p-values < 0.25 were considered significant. Permission must be obtained for use of copyrighted material from other sources (including the web). Please note that it is compulsory to follow figure instructions.

### 2.7 Differential expression of genes and protein-protein interaction analyses

We performed DEG analysis using the “limma” R package on the GSE17536, GSE14333, TCGA-COAD, and GTEx cohorts. Genes with an adjusted P-value < 0.05 and an absolute log2 fold change (FC) > 0.5 were identified as DEGs.

Protein-coding genes in the DEG were used to construct a PPI network using common transcripts, employing STRING with all parameters set to their default values (https://cn.string-db.org/). Subsequent Gene Ontology (GO) and Kyoto Encyclopedia of Genes and Genomes (KEGG) analyses were also performed through STRING using the DEGs.

### 2.8 Stromal and immune cells infiltration

The ESTIMATE algorithm was employed to identify the tumor microenvironment, and the ESTIMATE, immune, and stroma scores were calculated using the R software package “estimate” (18). The cellular composition of stromal and immune cells in the tumor within the GSE17536 dataset was estimated using the R software package “xCell” (19). Scores for immune and stromal cells were calculated for each sample. Additionally, the CIBERSORTx online platform (https://cibersortx.stanford.edu/) was utilized to assess the infiltration of 22 immune cell types in each sample (20).

### 2.9 Cell lines, antibodies, and chemicals

All cell lines were obtained from the Cell Bank of Shanghai, Institutes for Biological Sciences, China, and tested negative for mycoplasma infection. These cells were cultured in DMEM medium or RPMI 1640 medium (Thermo Fisher Scientific, Waltham, MA, USA), supplemented with 10% fetal bovine serum (Thermo Fisher Scientific, Waltham, MA, USA), at 37°C in a humidified atmosphere containing 5% CO2. 5% O2 hypoxic cell culture was performed by incubating cells in a sealed container with a Mitsubishi AnaeroPack™ anaerobic gas generator (Mitsubishi Gas Chemical Co., Tokyo, Japan). Hypoxic conditions were verified with the use of a Mitsubishi RT Anaero-Indicator (Mitsubishi Gas Chemical Co., Tokyo, Japan).

Antibodies against HIF1α and β-actin were purchased from Cell Signaling Technology (Danvers, MA, USA). Antibodies against ACTA2, ACTN1, CAVIN3, CEP170, LTBP1 and POSTN were purchased from Proteintech Group (Rosemont, IL, USA), Antibody against PCSK5 was purchased from CUSABIO (Wuhan, Hubei, China). Antibodies were diluted according to manufacture instructions.

### 2.10 Protein extraction and western blotting

The cells were washed with PBS and trypsinized, neutralization with serum-supplemented media, washed with PBS, and resuspended in RIPA buffer (Sigma-Aldrich, St. Louis, MO, USA). A 1% protease inhibitor cocktail (Halt™ Protease Inhibitor Cocktail, EDTA-Free, Thermo Fisher Scientific) was added to the mixture. The lysate was collected by centrifugation at 12,000 rpm at 4°C for 15 minutes. The supernatant was transferred to a new tube, and its concentration was determined using the BCA protein quantification assay. The supernatant was mixed with loading buffer (Sigma-Aldrich, St. Louis, MO, USA) and denatured by boiling at 95 °C.

Samples were subjected to SDS-PAGE gel electrophoresis, and proteins were subsequently transferred to PVDF membranes. The membranes were blocked with 5% non-fat milk in TBST and then incubated with specific antibodies overnight at 4°C with gentle agitation. Following washing, the membranes were incubated with HRP-conjugated secondary antibodies. Protein bands were visualized using chemiluminescent substrates.

### 2.11 Total RNA extraction and quantitative real-time PCR

Total RNA was extracted from cells using TRIzol Reagent (Takara, Kusatsu, Japan) following the manufacturer’s instructions. After assessing quality and quantity, samples were then stored at –80°C.

The extracted RNA was reverse-transcribed into cDNA using PrimeScript™ RT Master Mix (Takara, Kusatsu, Japan). The resulting cDNA was stored at –20°C for further analysis.

Gene expression levels were quantified using qRT-PCR with gene-specific primers and the One Step SYBR PrimeScript RT-PCR Kit II (Takara, Kusatsu, Japan). The qRT-PCR reaction conditions followed the manufacturer’s instructions. Expression levels were normalized to β-actin, and relative quantification was performed using the 2^-ΔΔCt method.

### 2.12 siRNA-mediated RNA interference

Two siRNAs for each targeting human PCSK5 and POSTN (designated as si-PCSK5_1, si-PCSK5_2, si-POSTN_1, and si-POSTN_2) and a nontargeting control siRNA were purchased from RiboBio (Guangzhou, Guangdong, China). The siRNA target sequences were as follows: si-PCSK5_1: GCAAGTACGGATTCATCAA, si-PCSK5_2: CGGGACATTTGAACGCTAA, si-POSTN_1: GCACTTGTAAGAACTGGTA, and si-POSTN_2: GCTCAGAGTCTTCGTATAT. For transfection, Lipofectamine 3000 (Invitrogen, Carlsbad, CA, USA) was used according to the manufacturer’s instructions. After 48 hours, some of the cells were harvested for Western blot analysis to assess the effects of siRNA inhibition.

### 2.13 *In vitro* migration assay

Cell migration was assessed using Transwell chambers (Corning, NY, USA). Suspensions of 10 × 10^4 cells in 200 µL of serum-free medium were added to the upper chamber, while the lower chamber contained medium with 10% FBS. After 16–24 hours, the cells were washed with PBS and fixed in 4% paraformaldehyde. The cells on the upper polycarbonate membranes were gently wiped with cotton swabs. The migrating cells were stained with crystal violet and then counted in four random fields under a light microscope.

### 2.14 Statistical analysis

Statistical analysis was conducted using R software. Forest plots were generated using univariate or multivariate Cox proportional hazard regression to calculate the hazard ratio (HR). The Kaplan-Meier method was employed for survival analysis. The Wilcoxon test was used to assess differences between groups. Statistically significant differences were indicated as follows: *p < 0.05; **p <0.01; ***p < 0.001; NS indicates not significant.

## 3 Results

### 3.1 Hypoxia is an important prognostic factor in patients with colorectal cancer

**Figure 1:**
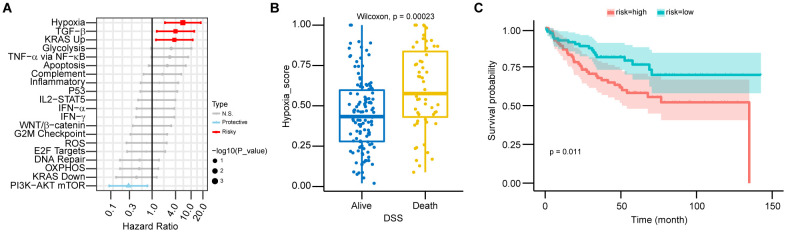
Hypoxia as a major prognostic factor in CRC patients. **(A)** Forest plot showing hazard ratios (HR) from univariate Cox regression for 20 cancer hallmark pathways in CRC patients. HRs and 95% confidence intervals (CIs) were calculated, with statistical significance assessed by the Wald test (p-value). Pathways with p > 0.05 are labeled “N.S.” (not significant), HR > 1 as “Risky,” and HR < 1 as “Protective.” Error bars represent 95% CIs. **(B)** The boxplot shows the distribution of risk scores across DSS groups in CRC patients, including median values and interquartile ranges (IQR). Statistical significance was assessed using the Wilcoxon test (p < 0.01), with higher hypoxia scores associated with worse survival outcomes. **(C)** Kaplan-Meier survival curves for high-risk and low-risk CRC patients, stratified by risk score. Statistical significance was assessed by the log-rank test (p < 0.05), with higher hypoxia scores associated with poorer prognosis.

RNA-seq data from the GSE17536 dataset were utilized to calculate the ssGSEA scores for cancer hallmark pathways. Significant associations with prognosis were observed for hypoxia (HR: 6.14, 95% CI: 2.13–17.67, p = 0.001), TGF-β pathway (HR: 3.98, 95% CI: 1.32–12.01, p = 0.014), KRAS upregulation pathway (HR: 3.73, 95% CI: 1.26–11.03, p = 0.017), and PI3K-AKT mTOR pathway (HR: 0.25, 95% CI: 0.08–0.76, p = 0.014) (**Figure 1A**). Patients were categorized into two groups based on their prognosis. Those with a worse prognosis exhibited higher hypoxia scores (p < 0.001) (**Figure 1B**). Subsequently, patients were stratified into high-risk and low-risk groups using the median hypoxia score as the threshold. The high-risk group demonstrated significantly poorer survival (p = 0.011) (**Figure 1C**).

### 3.2 Construction of a hypoxia-related score using WGCNA clustering and LASSO regression model to predict the prognosis of colorectal cancer patients

**Figure 2:**
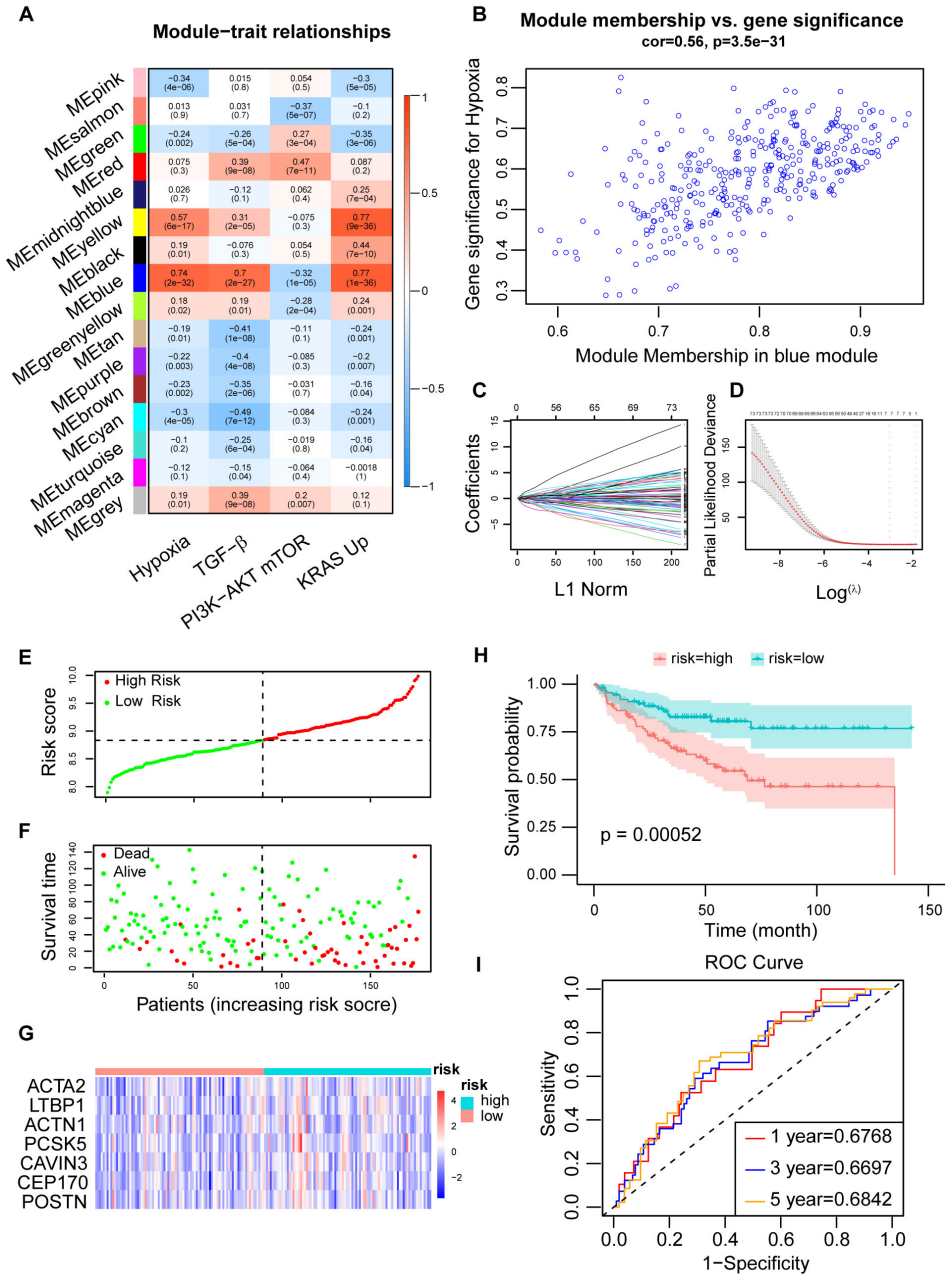
Construction of CRC prognosis prediction model using LASSO regression. **(A)** Determination of the correlation between modules and prognostic cancer hallmarks, including hypoxia, using module-trait correlation analysis. Correlations between module eigengenes (MEs) and cancer hallmarks were visualized using a heatmap. Pearson correlation coefficients were calculated between MEs and cancer hallmarks, and the corresponding p-values were obtained using the Student’s t-test. **(B)** Correlations between genes in the blue module and hypoxia, with gene module membership (GMM) and gene trait significance (GTS) calculated for hypoxia-related traits. **(C)** LASSO coefficient profiles for hypoxia-related prognostic differential expressed genes. The coefficient values for the selected genes were plotted against the penalty parameter (lambda). **(D)** Cross-validation curve for the LASSO regression model, used to determine the optimal penalty parameter (lambda) for prognostic gene selection. **(E, F)** Distribution of the Hypoxia-related Prognostic Score (HPS) which was calculated based on the expression of seven hypoxia-related signature genes across CRC patients, along with their survival status and survival time showing worse prognosis following higher HPS. **(G)** Expression profiles of the seven signature genes in high- and low-risk patient groups, stratified by their HPS and visualized using a heatmap. **(H)** Kaplan-Meier survival curve for CRC patients stratified into high-risk and low-risk groups based on their HPS. Statistical significance was assessed by the log-rank test (p < 0.01). **(I)** Time-dependent ROC curves for the HPS at 1-, 3-, and 5-year time points in the training dataset (GSE17536). The area under the curve (AUC) values indicated the prognostic performance of the HPS.

Using WGCNA, genes were categorized into 16 modules based on their correlation, with the blue module exhibiting the strongest association with hypoxia **(Figures 2A, B**; **Supplementary Figures 1A, B**). Univariate Cox analysis identified genes linked to patient prognosis, and LASSO regression analysis subsequently pinpointed 7 genes of interest (ACTA2, ACTN1, CAVIN3, CEP170, LTBP1, PCSK5, and POSTN). Based on the expression levels of these genes, we developed a novel hypoxia-related prognostic score (HPS) (**Figures 2C, D**). Patients were stratified into two groups using the median HPS, revealing significant differences in prognosis (p < 0.001), with the high-risk group faring worse. The distribution of HPS also varied significantly among patients with different prognoses (p < 0.001) (**Figures 2E–H**; **Supplementary Figure 1C**). Analysis of the 7 HPS signature gene expressions in colorectal cancer tumors versus normal tissues, using TCGA and GTEx databases, showed a marked difference (**Supplementary Figure 1D**). The ROC curves for 1-, 3-, and 5-year overall survival (OS) based on HPS yielded areas under the curve of 0.6768, 0.6697, and 0.6842, respectively (**Figure 2I**).

### 3.3 Combining clinicopathological features with HPS to construct a nomogram predicting the prognosis of CRC patients

**Figure 3:**
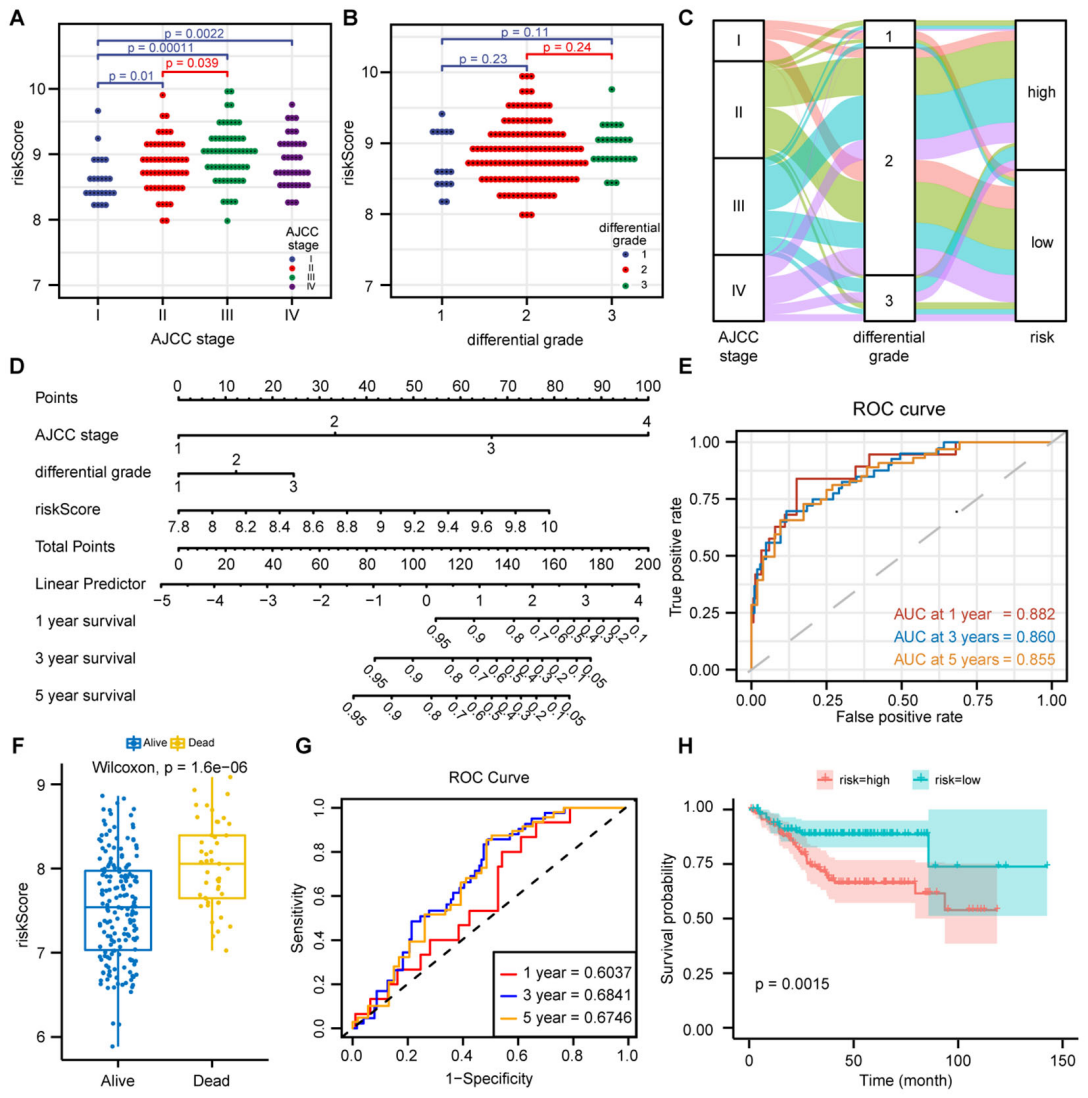
Prediction of CRC prognosis by combining clinicopathological features with HPS. **(A)** Distribution of Hypoxia-related Prognostic Score (HPS) across AJCC stages I-IV. Statistical analysis (Wilcoxon test, p < 0.05) revealed significantly higher HPS in advanced stages. **(B)** Distribution of HPS across different differentiation grades (well-differentiated, moderately differentiated, and poorly differentiated) in CRC patients. Data are presented as dot plots, with statistical significance assessed by the Wilcoxon test. **(C)** Alluvial diagram showing the relationships between AJCC stage, differentiation grade, and HPS risk group. **(D)** Nomogram integrating clinicopathological features and HPS for predicting 1-, 3-, and 5-year overall survival (OS) in CRC patients, based on a multivariate Cox regression model. **(E)** Time-dependent ROC curves of the nomogram at 1-, 3-, and 5-year time points in the GSE17536 training dataset, with AUC values indicating prognostic performance. **(F)** Boxplot of HPS distribution in different prognosis groups in the GSE14333 validation dataset. Wilcoxon test (p < 0.01) showed higher HPS in poor prognosis groups. **(G)** Time-dependent ROC curves of HPS at 1-, 3-, and 5-year time points in the GSE14333 dataset, with AUC values assessing HPS’s prognostic accuracy. **(H)** Kaplan-Meier survival curves for high-risk and low-risk groups based on HPS in GSE14333. Log-rank test (p < 0.01) showed significantly worse survival in the high-risk group.

The distribution of HPS in CRC patients varied across different AJCC clinical stages, with later stages showing higher HPS (**Figures 3A, B**). The distribution of HPS in CRC patients with various clinicopathological characteristics is depicted in alluvium plots (**Figure 3C**). The study created a nomogram that integrates HPS with clinicopathological characteristics to predict prognosis (**Figure 3D**). The areas under the ROC curves for 1-, 3-, and 5-year OS of nomogram in CRC were 0.882, 0.860, and 0.855, respectively (**Figure 3E**). The nomogram predicted outcomes were largely consistent with the actual outcomes (**Supplementary Figure 1E**). To confirm the predictive capability of this score, the study applied HPS to forecast the prognosis of CRC patients in the validation set GSE14333. The distribution of HPS among patients with different prognoses was distinct (p < 0.001), and a statistically significant difference in prognosis was noted between the high- and low-risk HPS groups (p = 0.0015) (**Figures 3F, H**). The areas under the ROC for 1-, 3-, and 5-year OS of HPS were 0.6037, 0.6841, and 0.6746, respectively (**Figure 3G**).

### 3.4 Hypoxia changes HPS signature gene expression in normal intestinal epithelial cells and CRC cells in different patterns

**Figure 4:**
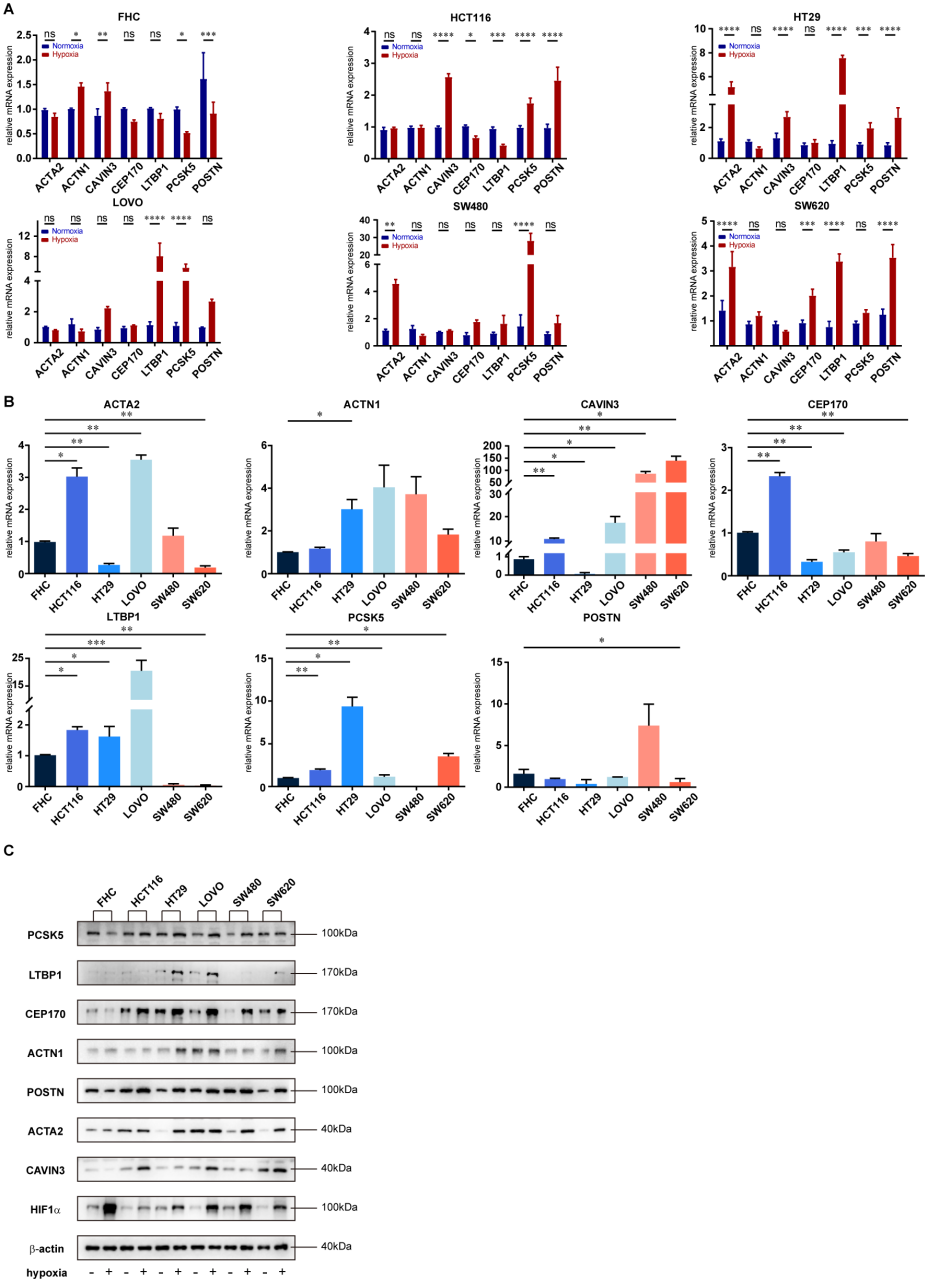
Different impacts of hypoxia on the expression of HPS signature genes in normal intestinal epithelial and CRC cells. **(A)** RT-PCR analysis of mRNA expression changes in HPS signature genes under hypoxic conditions in FHC normal intestinal epithelial cells and five colorectal cancer (CRC) cell lines (HCT116, HT29, LOVO, SW480, SW620). Total RNA was extracted from cells exposed to hypoxia. Data are presented as mean with error bars representing standard deviation (SD) from three biological replicates. Statistical significance was assessed using ANOVA, with *p < 0.05; **p < 0.01; ***p < 0.001; ****p < 0.0001; ns, not significant. **(B)** Expression profiles of HPS signature genes in FHC and CRC cell lines (HCT116, HT29, LOVO, SW480, SW620) following hypoxia. Data are presented as mean with error bars representing SD from three biological replicates. Statistical significance was assessed using ANOVA, with *p < 0.05; **p < 0.01; ***p < 0.001 indicating significant differences between groups. **(C)** Western blot analysis of protein expression changes in HPS signature genes following hypoxia in FHC and CRC cell lines. Cells were exposed to hypoxia, and protein lysates were analyzed by western blotting.

To further investigate the molecular mechanisms of hypoxia regulation in the CRC TME, we treated human normal intestinal epithelial cells (FHC) and five human CRC epithelial cell lines (HCT116, HT-29, LOVO, SW480, and SW620) with hypoxia *in vitro*. We analyzed HIF1α protein expression via western blot to confirm the successful construction of the hypoxia model (**Supplementary Figure 2A**). Subsequently, we conducted qRT-PCR on the hypoxic cell lines to assess the expression of seven HPS signature genes (ACTA2, ACTN1, CAVIN3, CEP170, LTBP1, PCSK5 and POSTN). We observed that ACTN1 and CAVIN3 were slightly upregulated in FHC cells post-hypoxia, while the CRC cell lines exhibited varying degrees of upregulation, which was more pronounced than in FHC cells (**Figure 4A**). The marked disparity in gene expression changes between FHC and CRC cell lines following hypoxia indicates that the responses of normal intestinal epithelial cells and CRC cell lines to TME hypoxia are distinct (**Supplementary Figure 2B**). Further analysis of gene expression in both normal intestinal epithelial cell lines and tumor cell lines under normoxic conditions revealed significant differences in signature gene expression patterns between FHC and CRC cell lines (**Figure 4B**).

We conducted Western blot analysis to further explore the differences in protein expression between FHC and CRC cell lines under hypoxic conditions. The results indicated that the protein levels of all signature genes were altered following hypoxia, showing a high degree of consistency with our qRT-PCR findings (**Figure 4C**). Additionally, after knocking down POSTN and PCSK5 in CRC cell lines (HCT116 and LOVO) using siRNAs, our *in vitro* cell migration assays demonstrated that both genes are involved in the migration of CRC cells (**Supplementary Figures 2C–E**).

### 3.5 Functional enrichment analysis of differentially expressed genes identified by HPS risk model

**Figure 5:**
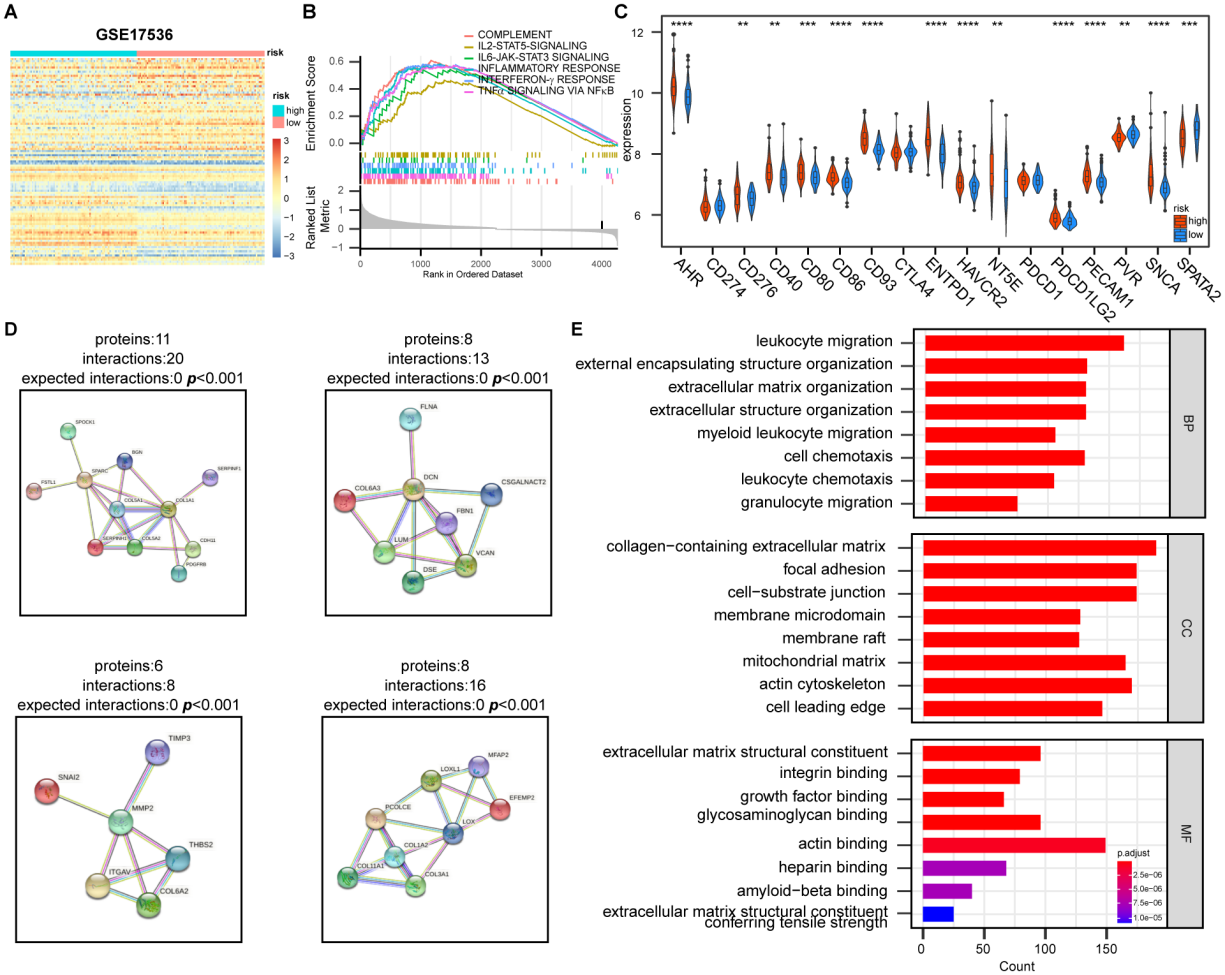
Effect of hypoxia on the expression of genes is related to tumor immune response and extracellular matrix construction. **(A)** Heatmap of differentially expressed genes between high- and low-risk groups based on HPS. Genes were selected based on fold change and statistical significance. **(B)** Gene Set Enrichment Analysis (GSEA) of immune-related pathways in high- and low-risk groups, showing significantly enriched pathways. **(C)** Differential expression of immunotherapy-related genes between high- and low-risk groups. Statistical significance was assessed using the t-test with FDR adjustment (**p < 0.01, ***, p < 0.001, ****, p < 0.0001). **(D)** The four most significantly altered protein-protein interaction (PPI) networks, identified using STRING database (https://cn.string-db.org/), highlighting key interactions between differentially expressed genes. **(E)** Gene Ontology (GO) functional enrichment analysis of the PPI network, showing the most enriched biological processes, molecular functions, and cellular components.

After differential gene expression analysis between high- and low-risk groups and a GSEA enrichment analysis revealed that, in addition to the hypoxia pathway (p < 0.001), immune-related pathways such as inflammatory response (p < 0.001), interferon-γ response (p < 0.001), complement pathway (p < 0.001), and NF-κB-mediated TNF-α pathway (p < 0.001) were significantly enriched in the high-risk group (**Figures 5A, B**; **Supplementary Figure 3A**). Significant differences were observed in the expression patterns of genes related to immunotherapy among DEGs, although no significant differences were detected in the expression of the immune checkpoint inhibitor (ICI) genes CD274, PDCD1, and CTLA-4 (**Figure 5C**; **Supplementary Figure 3B**).Furthermore, the protein-protein interaction (PPI) analysis and GO enrichment analysis of protein-coding genes with the most significant changes in DEGs indicated that pathways were primarily enriched in the migration and chemotaxis of immune cells and the composition and structure of extracellular matrix (**Figures 5D, E**; **Supplementary Figure 3C**), suggesting that hypoxia has an influence on immune cell migration, thereby affecting the TME.

### 3.6 HPS risk is negatively related with immune response in TME

**Figure 6:**
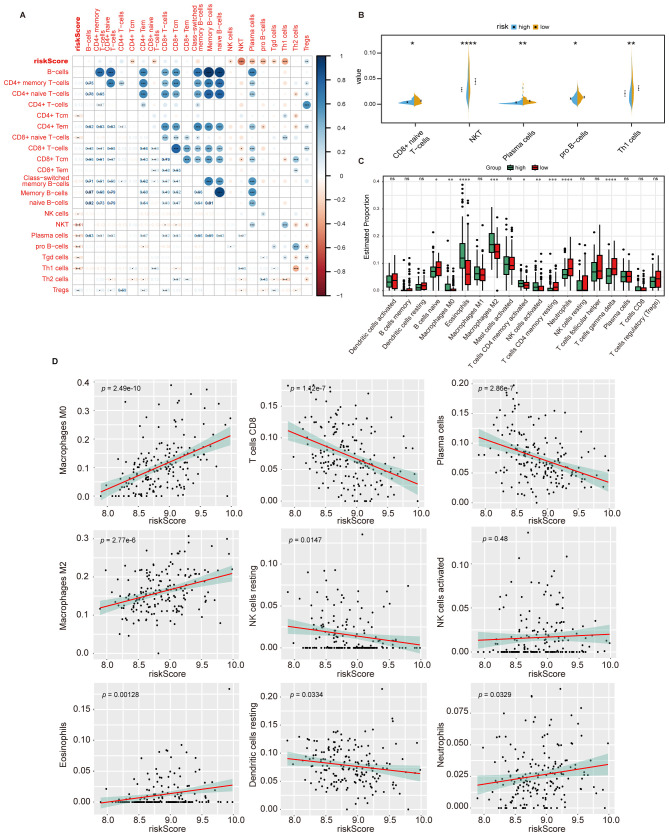
HPS is correlated with TME components. **(A)** Correlation between HPS and the infiltration of lymphoid-derived immune cells, assessed using the xCell algorithm. The correlation coefficients are shown, with statistical significance indicated by asterisks (*, p < 0.05, **, p < 0.01, ***, p < 0.001). **(B)** Infiltration of lymphoid-derived immune cells in high- and low-risk groups, analyzed using the t-test. Statistical significance is indicated by asterisks (*, p < 0.05, **, p < 0.01, ***, p < 0.001, ****, p < 0.0001). **(C)** Immune cell infiltration in high- and low-risk groups as determined by CIBERSORT. Statistical significance was assessed using the Kruskal-Wallis test, with p-values indicated as: *p < 0.05; **p < 0.01; ***p < 0.001; ****p < 0.0001; ns, not significant. Error bars represent the interquartile range (IQR). **(D)** Linear correlation between HPS and immune cell infiltration levels in the tumor microenvironment as determined by CIBERSORT. Statistical significance was assessed using Pearson’s correlation test, with p-values indicating the strength of the correlation.

To discover how HPS risk is correlated with tumor immune, we performed xCell analysis to assess the infiltration of non-cancer cells in the TME, and its correlation with HPS was examined. In the analysis of different cell subsets, HPS showed a positive correlation with myeloid-derived immune cells (**Supplementary Figure 4A**) and a negative correlation with lymphoid-derived immune cells (**Figure 6A**). HPS was also positively correlated with most stromal cells and associated with other stem cells and some other cell types (**Supplementary Figures 4B, C, D**). The distribution of myeloid-derived immune cells, lymphoid-derived immune cells, and stromal cells between the high- and low-risk groups were further analyzed, revealing fewer myeloid-derived immune cells and stromal cells in the low-risk group, while lymphoid-derived immune cells were more abundant (**Figure 6B**, **Supplementary Figures 4E, F**). Additional analysis of immune cell infiltration using CIBERSORT indicated that macrophage infiltration predominated in the microenvironment (**Supplementary Figures 5A, B**). Notably, infiltration by undifferentiated macrophages and M2 macrophages significantly decreased in the low-risk group (**Figure 6C**, **Supplementary Figure 5C**). The linear correlation analysis between HPS and immune cell infiltration demonstrated that the hypoxia score was significantly positively correlated with undifferentiated macrophages but negatively correlated with the infiltration of cytotoxic CD8 + T cells and plasma cells (**Figure 6D**).

## 4 Discussion

Tumors require significant amounts of oxygen and nutrients to support their rapid proliferation. However, due to insufficient tumor blood vessel density and dysfunctional vascular structure, tumor cells are often in a hypoxia state. To progress, tumor cells evolve various mechanisms to adapt to hypoxic environments, involving alterations in metabolic pathways, regulation of gene expression, as well as interactions with other cells or tissues (2, 21–24). In this study, through a series of bioinformatics analyses, we found seven hypoxia-associated signature genes with the most significant prognostic impact on colorectal cancer patients and established a hypoxia-related prognosis score (HPS) for CRC based on this signature gene set. *In vitro*, we observed significant differences in the signature gene expression among normal and cancer cell lines under hypoxia condition, which confirmed the malignant predictive value of this HPS model.

TME hypoxia could mediate immune evasion of cancer cells via multiple mechanisms. Hypoxia has been shown to modulate the expression of cytokines and effector molecules of immune cells, inhibiting their cytotoxic function (13, 14, 21, 25). In this study, HPS was showed to be associated with immune cytokine pathways in tumor microenvironment. However, the current study found no significant difference in the expression of immunotherapy-related molecules PD-1/PD-L1 or CTLA-4 between the low HPS and high HPS group, which suggested that high HPS might not directly promote immunosuppression by altering expression of ICI-related molecules. Hypoxia may mediate the infiltration and distribution of immune cells in the TME by affecting their migration and chemotaxis and there is an inverse relationship between the degree of hypoxia and CD8+ T cell infiltration (16). In our study, HPS was found positively correlated to MDSCs and negatively correlated to NK cells and tumor killing T cells, suggesting that different HPS groups might have different immune cell infiltration thereby leading to an immune suppression environment. Further CIBERSORT infiltration analysis showed that high HPS group exhibited higher overall immune cell infiltration and this increase was predominantly observed in suppressive immune cell subsets such as Treg cells and MDSCs. Meanwhile, the infiltration of tumor killing immune cells such as NK cells and γδT cells was found decreased. All these findings emphase our hypoxia-related prognosis score denoted an immunosuppressive microenvironment for tumor.

Hypoxia may also indirectly alter immune cell infiltration by regulating the composition of the extracellular matrix (25, 26). Our PPI data revealed significant differences in collagen gene expression across different HPS groups, which supported the role of hypoxia in matrix regulation. Previous studies have discovered that macrophage infiltration contributes to the remodeling of the extracellular matrix (27). Through CIBERSORT analysis, we found increase macrophage infiltration in high HPS group compared to low HPS group, suggesting the immunosuppressive microenvironment in high HPS group might be related to extracellular matrix modification. In addition, both our findings and earlier researches support that alterations in the extracellular matrix composition could affect T-cell entrapment and function leading to immunosuppression (27).

Our study confirmed the significant impact of hypoxia on CRC outcomes via transcriptomic analysis. Notably, it suggested that the unique effects of hypoxia on the extracellular matrix and immune cell infiltration might lead to varying patient prognoses. Revealing the importance that hypoxia in the TME might contribute to a potential targeted approach, such as hyperbaric oxygen, to reverse tumor favoring TME. When combined with immunotherapy, reversing hypoxia could enhance outcomes for CRC patients.

## Data Availability

The datasets presented in this study can be found in online repositories. The names of the repository/repositories and accession number(s) can be found in the article/**Supplementary Material**.
