# Supplementary material for: Hypoxia reconstructed colorectal tumor microenvironment weakening anti-tumor immunity: construction of a new prognosis predicting model through transcriptome analysis
Source: Front Immunol. 2024 Dec 6;15:1425687. doi: 10.3389/fimmu.2024.1425687 (PMC11659140; doi:10.3389/fimmu.2024.1425687)
Supplement: Supplementary file 1 [file DataSheet1.zip › Supplementary_Material/Supplementary_Material.docx]

Supplementary Material

##
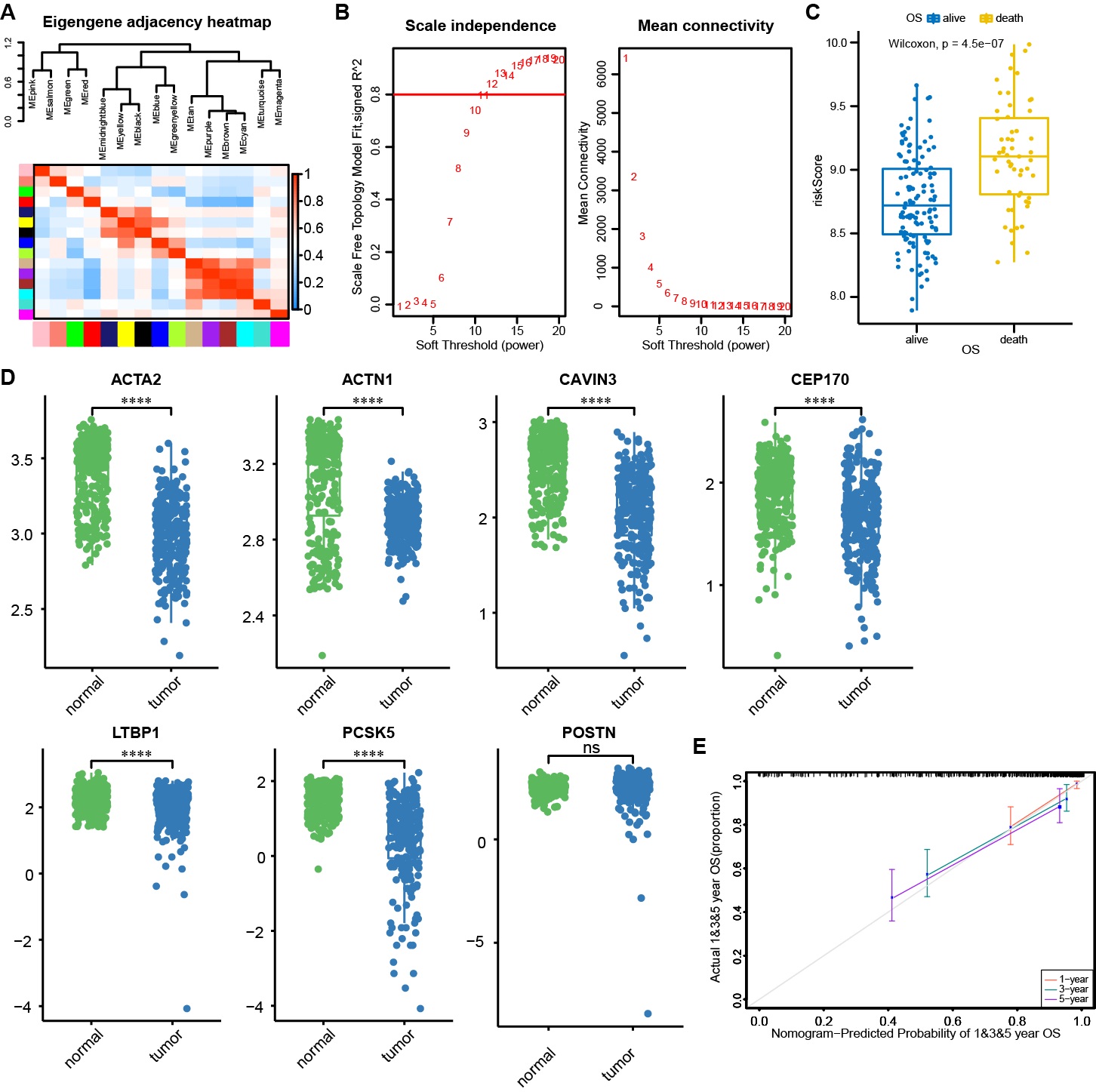
Supplementary Figures

**Supplementary Figure 1.** **Construction of CRC prognostic model using WGCNA and LASSO regression.**

(A) Heatmap showing the correlation coefficients between different gene modules.

(B) Selection of the optimal soft threshold for the WGCNA model.

(C) Distribution of Hypoxia-related Prognostic Score (HPS) in CRC patients from the GSE17536 dataset. Boxplot of HPS across survival groups, with median values and interquartile ranges (IQR). Statistical significance was assessed by the Wilcoxon test (p < 0.001).

(D) Differential expression of HPS signature genes in normal versus tumor colorectal tissues from TCGA and GTEx data. Statistical significance was assessed by the t-test (****, p < 0.001, ns: not significant).

(E) Comparison of Nomogram predictions for 1-, 3-, and 5-year survival outcomes with actual survival in CRC patients.


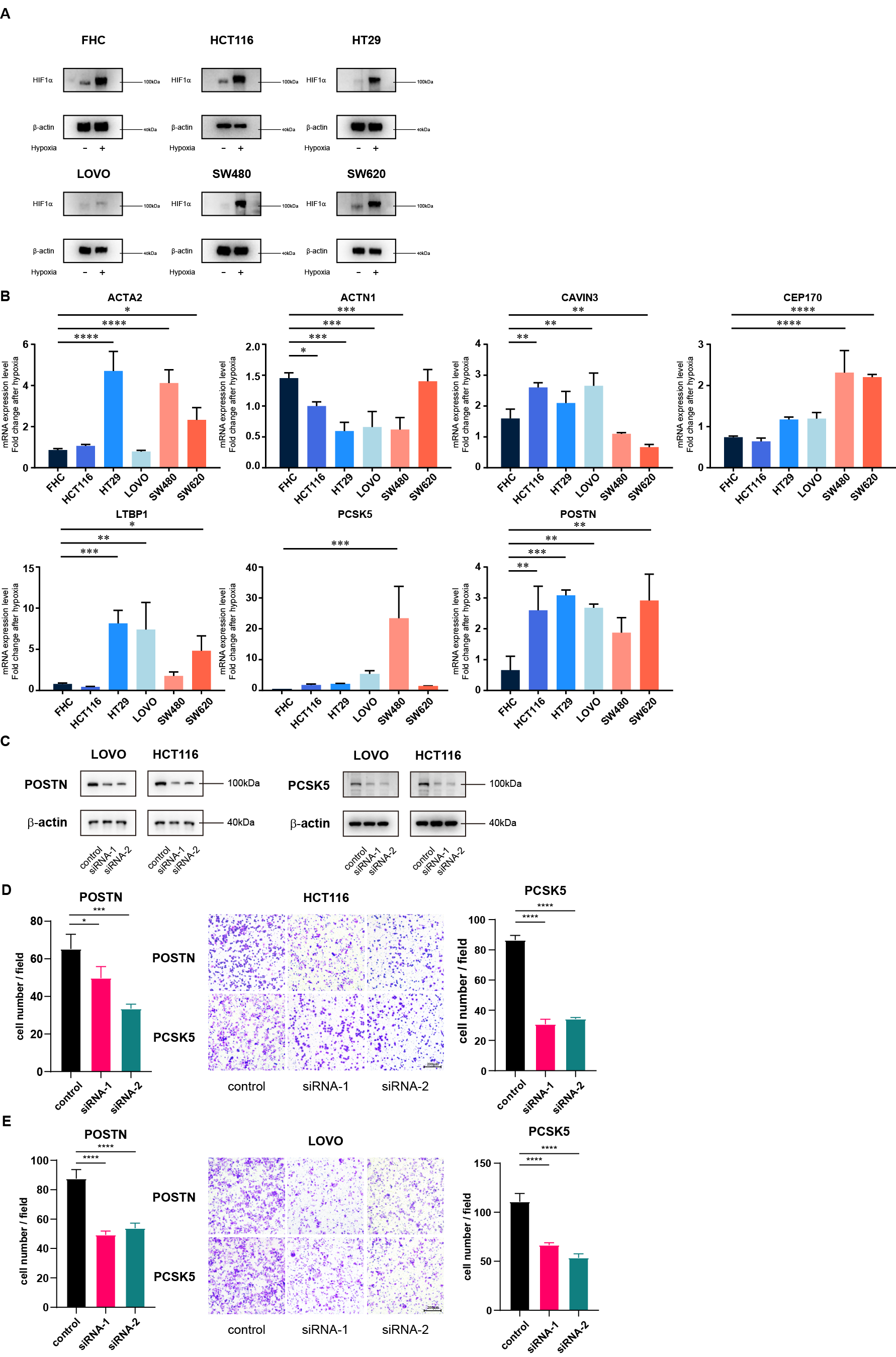


**Supplementary Figure 2. Impact of hypoxia on the expression of HPS signature genes in normal intestinal epithelial and CRC cells.**

(A) Western blot showing HIF1α protein expression after hypoxia exposure in FHC normal intestinal epithelial cells and five CRC cell lines (HCT116, HT29, LOVO, SW480, SW620).

(B) Changes in HPS signature gene expression after hypoxia exposure in FHC and CRC cell lines. Data are presented as mean with error bars representing SD from three biological replicates. Statistical significance was assessed by ANOVA (*, p < 0.05; **, p < 0.01; ***, p < 0.001).

(C) Decreased protein expression of POSTN and PCSK5 in LOVO and HCT116 CRC cell lines after siRNA-mediated knockdown.

(D, E) Transwell migration assays for POSTN or PCSK5 knockdown in LOVO and HCT116 cells. Statistical significance was assessed by the t-test (*, p < 0.05, **, p < 0.01, ***, p < 0.001, ****, p < 0.0001).


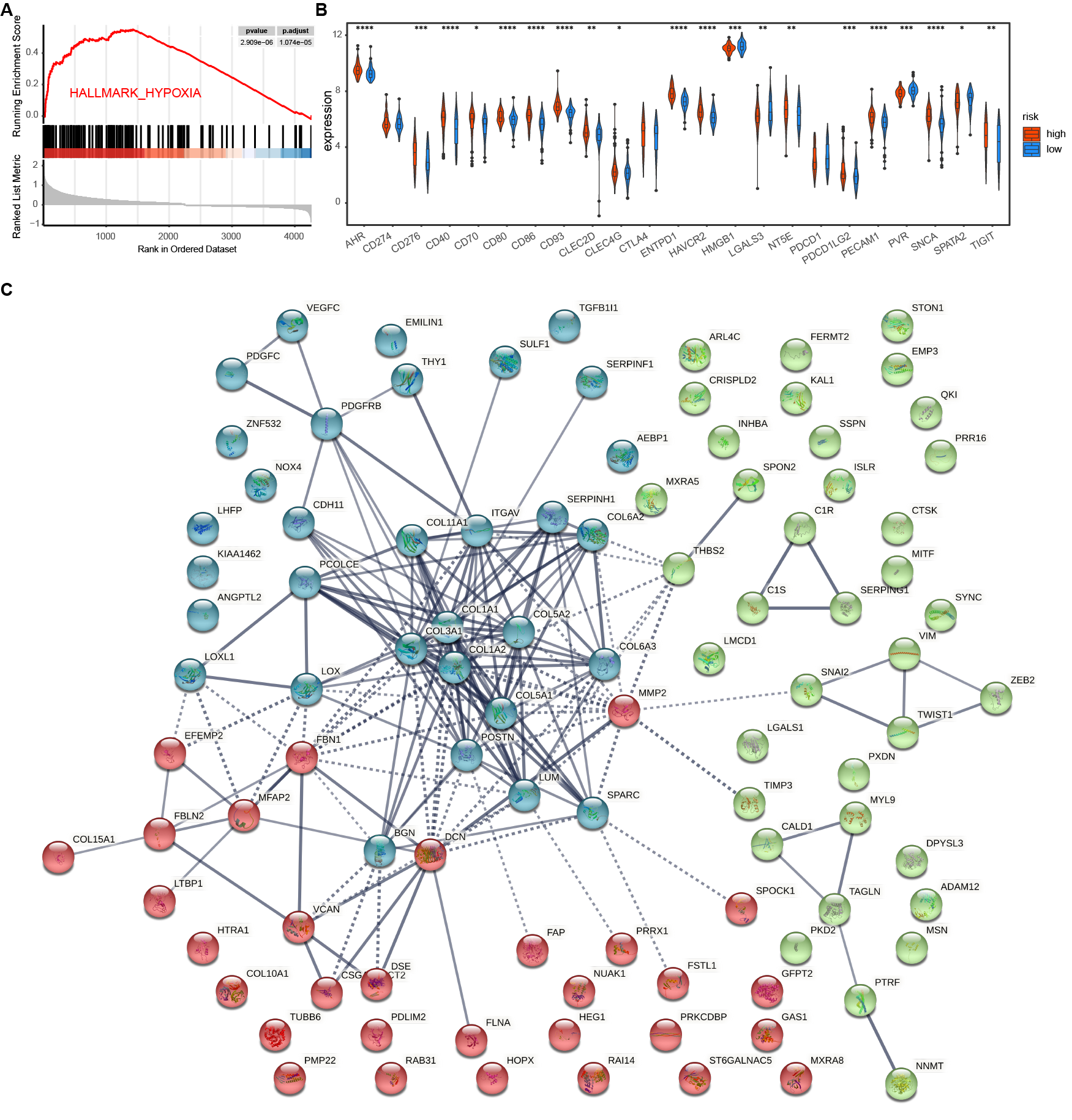


**Supplementary Figure 3.** **Differences in protein and gene expression in patients with different HPS risk groups.**

(A) Gene Set Enrichment Analysis (GSEA) of hypoxia-related pathways in high- and low-risk groups. Statistical significance was assessed by the Wilcoxon test.

(B) Expression differences of immunotherapy-related genes in the GSE14333 validation dataset. Statistical significance was assessed by the t-test (*, p < 0.05; **, p < 0.01; ***, p < 0.001; ****, p < 0.0001).

(C) Protein-protein interaction (PPI) network analysis of differentially expressed genes (DEGs).


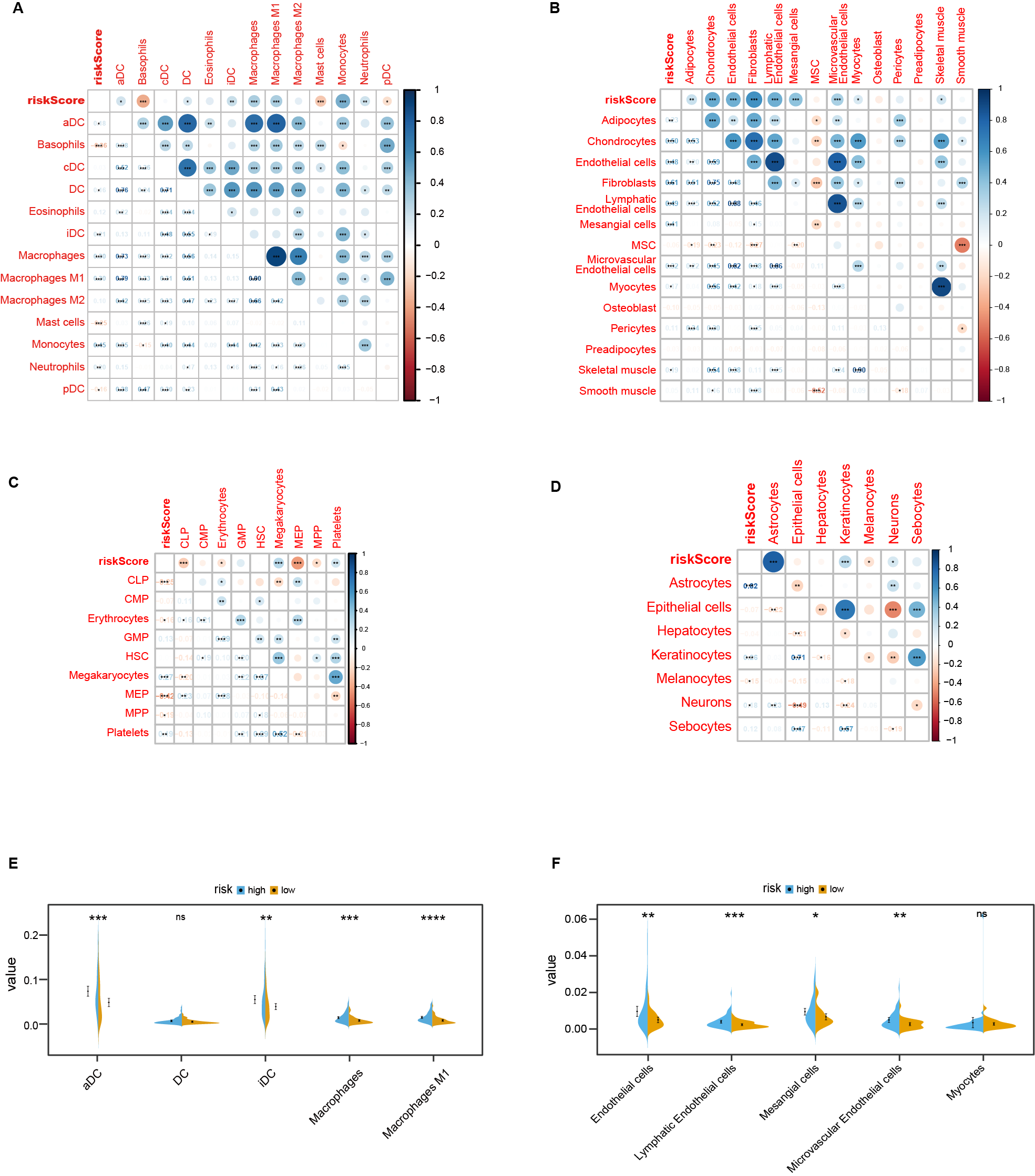


**Supplementary Figure 4.** **Correlation between HPS and TME components validated by different algorithms.**

(A-D) Correlation between HPS and the infiltration of different immune cell types in the TME, assessed using the xCell algorithm. Correlation coefficients are shown, with statistical significance indicated by asterisks (*, p < 0.05; **, p < 0.01; ***, p < 0.001).

(E-F) Infiltration of myeloid-derived immune cells and stromal cells in high- and low-risk groups, analyzed by the t-test. Statistical significance is indicated by asterisks (*, p < 0.05; **, p < 0.01; ***, p < 0.001; ****, p < 0.0001; ns, not significant).


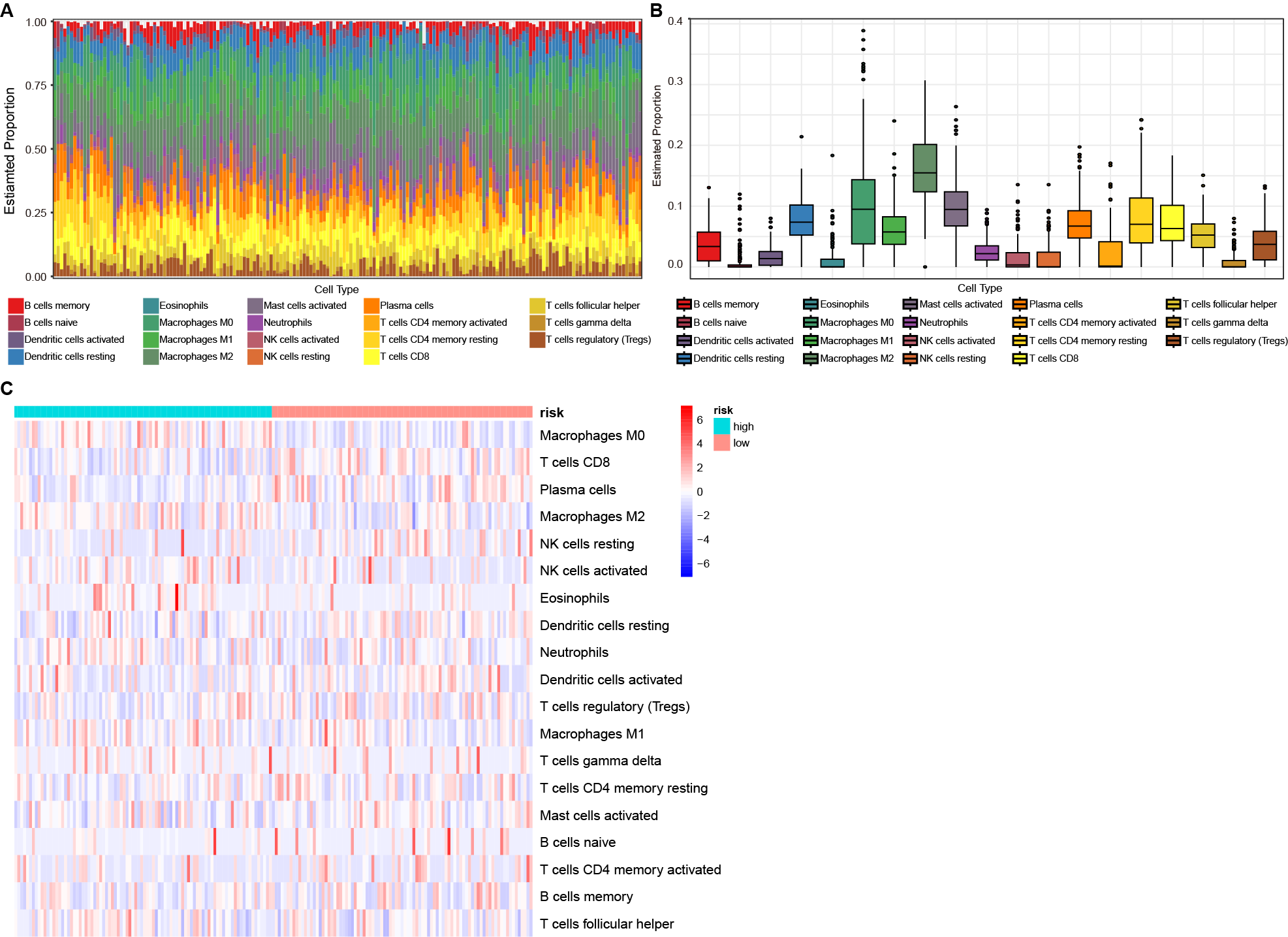


**Supplementary Figure 5.** **Immune cell infiltration in the CRC tumor microenvironment (TME) and differential infiltration between high- and low-risk groups.**

(A) Overall immune cell infiltration in CRC TME, presented as a stacked bar plot with immune cell proportions across samples, estimated using CIBERSORT.

(B) Individual immune cell infiltration in CRC TME, visualized using a boxplot.

(C) Differential immune cell infiltration between high- and low-risk groups, visualized using a heatmap.
