# Supplementary material for: Hypoxia reconstructed colorectal tumor microenvironment weakening anti-tumor immunity: construction of a new prognosis predicting model through transcriptome analysis
Source: Front Immunol. 2024 Dec 6;15:1425687. doi: 10.3389/fimmu.2024.1425687 (PMC11659140; doi:10.3389/fimmu.2024.1425687)
Supplement: Supplementary file 1 [file DataSheet1.zip › Supplementary_Material/Supplementary_Material.pdf]

Supplementary Material

1 Supplementary Figures

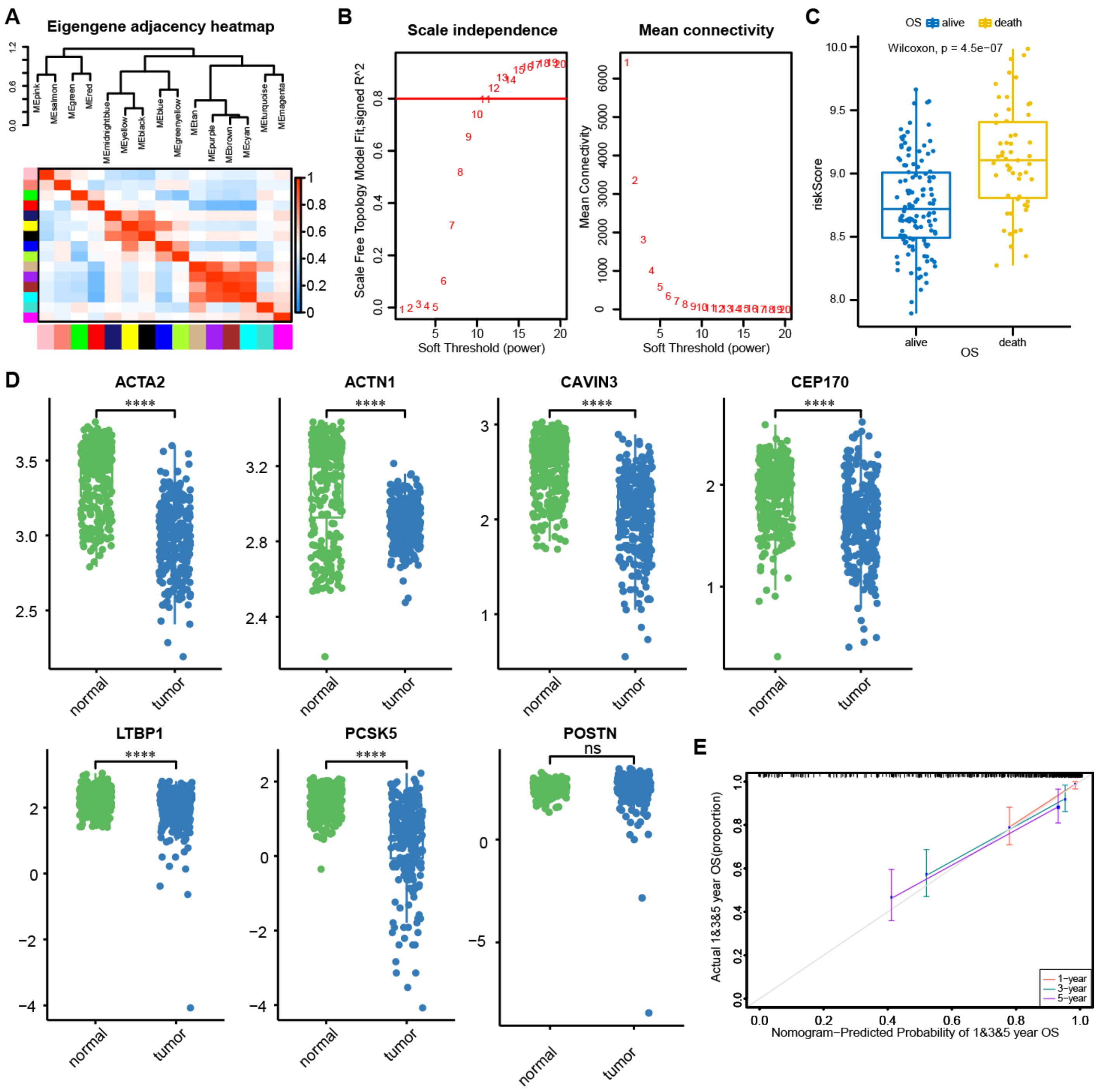

Supplementary Figure 1. Construction of CRC prognostic model using WGCNA and LASSO regression.

(A) Heatmap showing the correlation coefficients between different gene modules.

(B) Selection of the optimal soft threshold for the WGCNA model.

(C) Distribution of Hypoxia-related Prognostic Score (HPS) in CRC patients from the GSE17536 dataset. Boxplot of HPS across survival groups, with median values and interquartile ranges (IQR). Statistical significance was assessed by the Wilcoxon test ($p < 0.001$).

(D) Differential expression of HPS signature genes in normal versus tumor colorectal tissues from TCGA and GTEx data. Statistical significance was assessed by the t-test (****, $p < 0.001$, ns: not significant).

(E) Comparison of Nomogram predictions for 1-, 3-, and 5-year survival outcomes with actual survival in CRC patients.

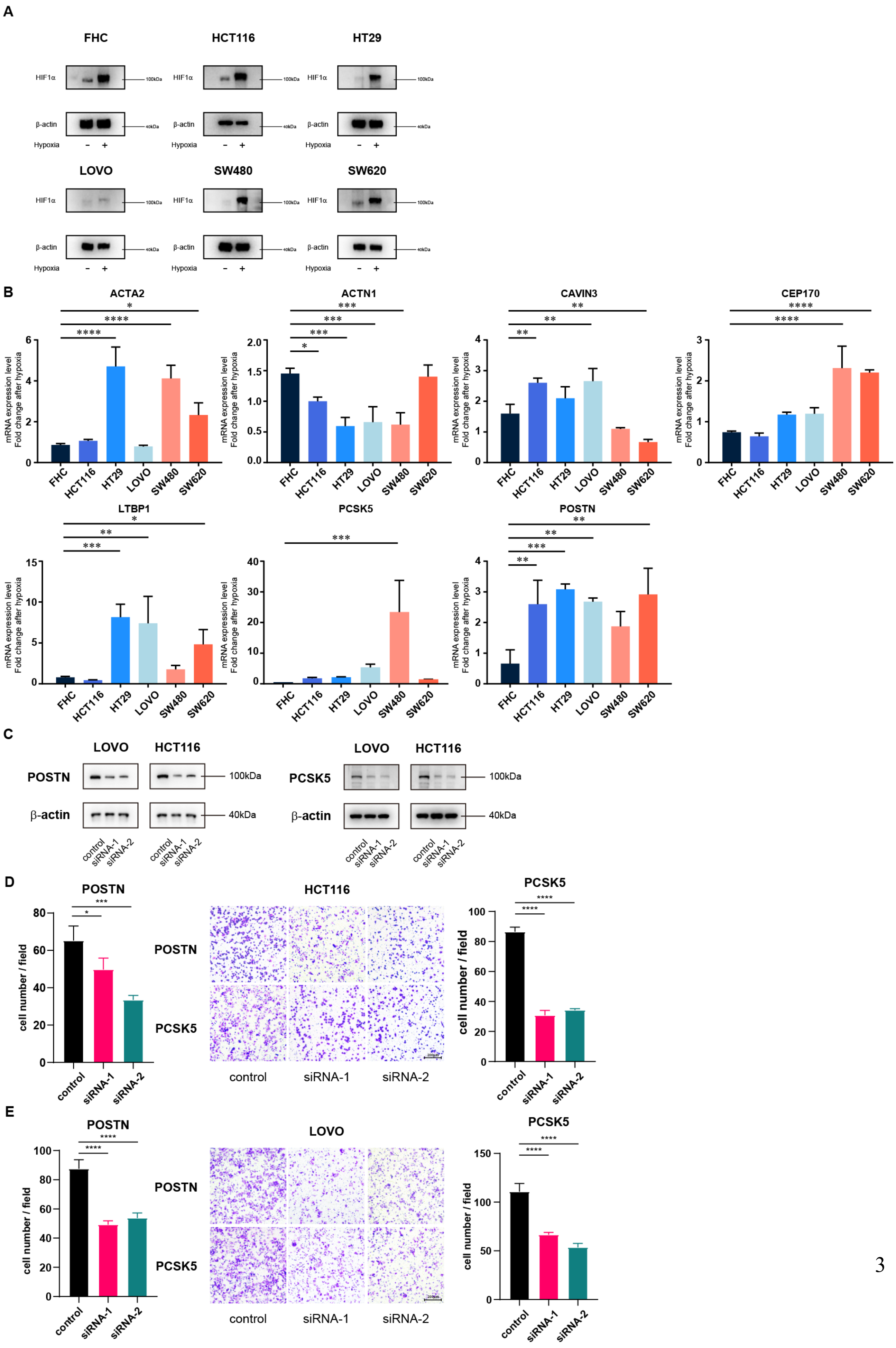
A
FHC
HCT116
HT29
LOVO
SW480
SW620
HIF1α
β-actin
Hypoxia
100kDa
40kDa
B
ACTA2
ACTN1
CAVIN3
CEP170
LTBP1
PCSK5
POSTN
mRNA expression level
Fold change after hypoxia
C
LOVO
HCT116
POSTN
PCSK5
β-actin
100kDa
40kDa
control
siRNA-1
siRNA-2
D
POSTN
HCT116
PCSK5
cell number / field
control
siRNA-1
siRNA-2
E
POSTN
LOVO
PCSK5
cell number / field
control
siRNA-1
siRNA-2

Supplementary Figure 2. Impact of hypoxia on the expression of HPS signature genes in normal intestinal epithelial and CRC cells.

(A) Western blot showing HIF1α protein expression after hypoxia exposure in FHC normal intestinal epithelial cells and five CRC cell lines (HCT116, HT29, LOVO, SW480, SW620).

(B) Changes in HPS signature gene expression after hypoxia exposure in FHC and CRC cell lines. Data are presented as mean with error bars representing SD from three biological replicates. Statistical significance was assessed by ANOVA (*, $p < 0.05$; **, $p < 0.01$; ***, $p < 0.001$).

(C) Decreased protein expression of POSTN and PCSK5 in LOVO and HCT116 CRC cell lines after siRNA-mediated knockdown.

(D, E) Transwell migration assays for POSTN or PCSK5 knockdown in LOVO and HCT116 cells. Statistical significance was assessed by the t-test (*, $p < 0.05$, **, $p < 0.01$, ***, $p < 0.001$, ****, $p < 0.0001$).

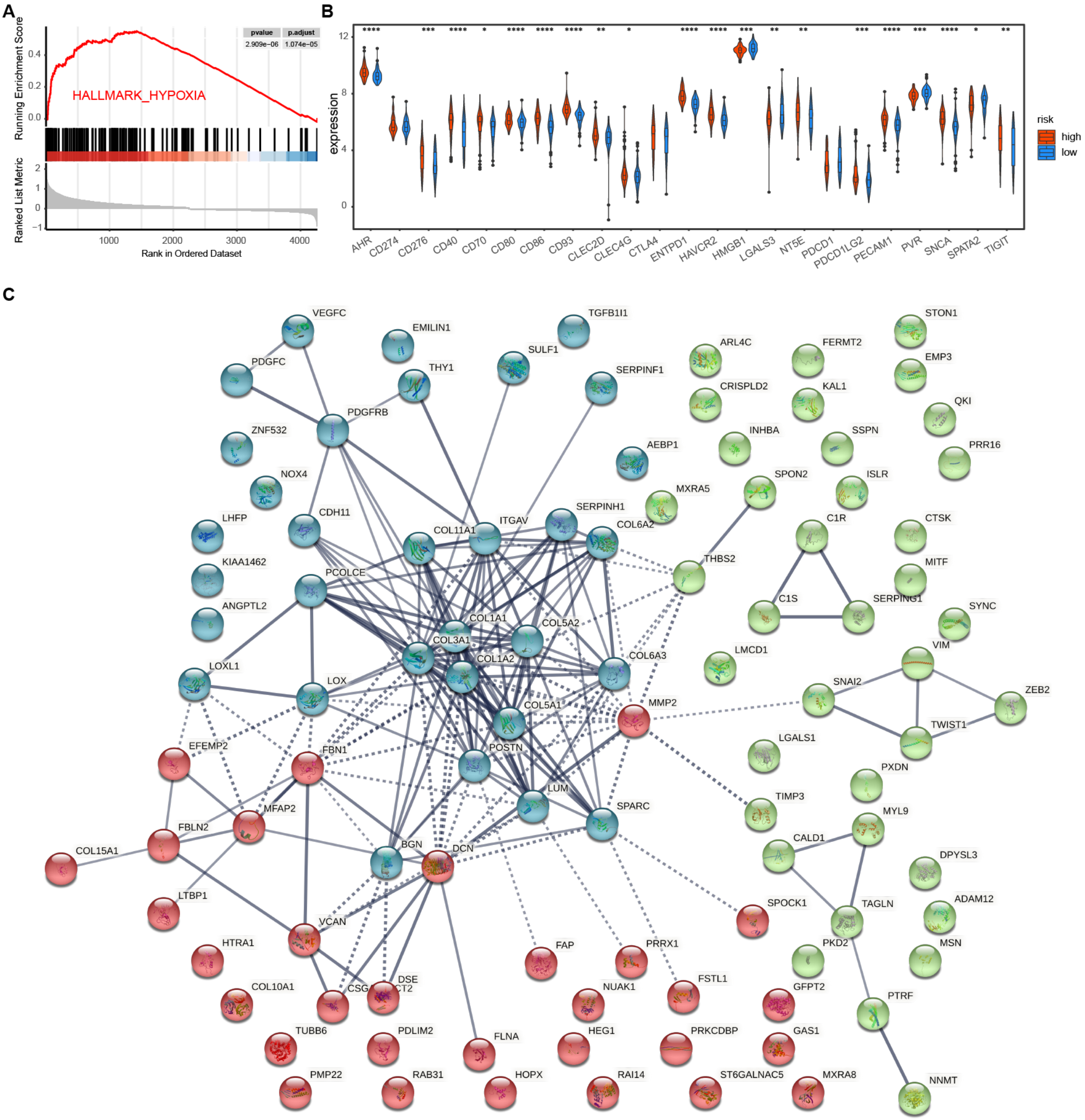
A
Running Enrichment Score
Ranked List Metric
Rank in Ordered Dataset
HALLMARK_HYPOXIA
pvalue
p.adjust
2.909e−06
1.074e−05
B
expression
risk
high
low
AHR
CD274
CD276
CD40
CD70
CD80
CD86
CD93
CLEC2D
CLEC4G
CTLA4
ENTPD1
HAVCR2
HMGB1
LGALS3
NT5E
PDCD1
PDCD1LG2
PECAM1
PVR
SNCA
SPATA2
TIGIT
C
VEGFC
EMILIN1
TGFB1I1
STON1
PDGFC
THY1
SULF1
SERPINF1
ARL4C
FERMT2
EMP3
CRISPLD2
KAL1
QKI
PDGFRB
ZNF532
INHBA
SSPN
PRR16
AEBP1
NOX4
SPON2
ISLR
MXRA5
CDH11
SERPINH1
LHFP
COL11A1
ITGAV
COL6A2
C1R
CTSK
KIAA1462
THBS2
MITF
PCOLCE
ANGPTL2
C1S
SERPING1
SYNC
COL1A1
COL5A2
COL3A1
VIM
COL1A2
COL6A3
LMCD1
LOXL1
LOX
SNAI2
ZEB2
MMP2
COL5A1
TWIST1
EFEMP2
FBN1
POSTN
LGALS1
PXDN
LUM
MFAP2
TIMP3
SPARC
MYL9
FBLN2
CALD1
COL15A1
BGN
DCN
DPYSL3
LTBP1
SPOCK1
TAGLN
ADAM12
VCAN
FAP
HTRA1
PRRX1
PKD2
MSN
DSE
COL10A1
NUAK1
FSTL1
GFPT2
PTRF
TUBB6
PDLIM2
FLNA
HEG1
PRKCDBP
GAS1
PMP22
RAB31
HOPX
RAI14
ST6GALNAC5
MXRA8
NNMT

Supplementary Figure 3. Differences in protein and gene expression in patients with different HPS risk groups.

(A) Gene Set Enrichment Analysis (GSEA) of hypoxia-related pathways in high- and low-risk groups. Statistical significance was assessed by the Wilcoxon test.

(B) Expression differences of immunotherapy-related genes in the GSE14333 validation dataset. Statistical significance was assessed by the t-test (*, $p < 0.05$; **, $p < 0.01$; ***, $p < 0.001$; ****, $p < 0.0001$).

(C) Protein-protein interaction (PPI) network analysis of differentially expressed genes (DEGs).

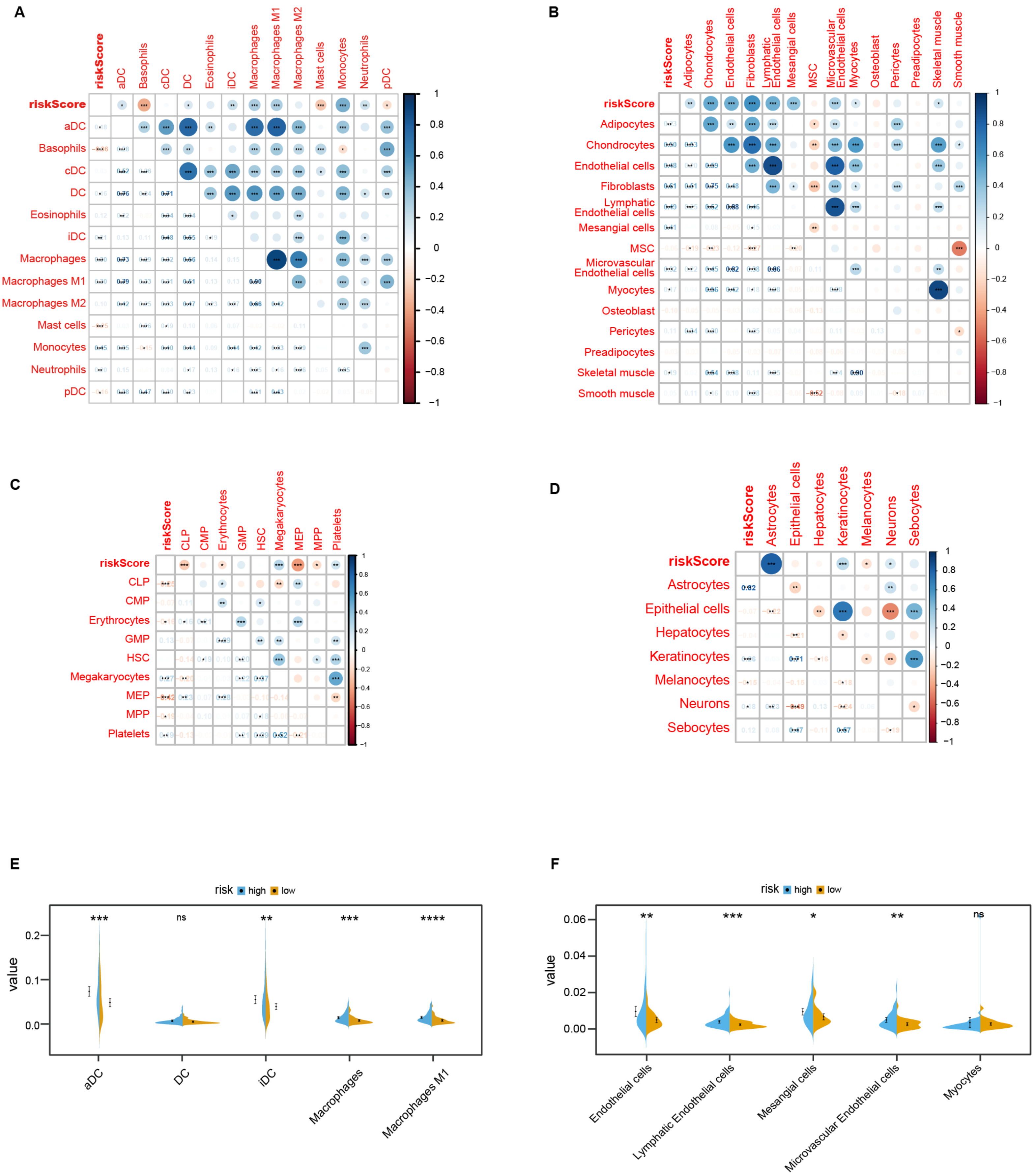
A
riskScore
aDC
Basophils
cDC
DC
Eosinophils
iDC
Macrophages
Macrophages M1
Macrophages M2
Mast cells
Monocytes
Neutrophils
pDC
B
riskScore
Adipocytes
Chondrocytes
Endothelial cells
Fibroblasts
Lymphatic Endothelial cells
Mesangial cells
MSC
Microvascular Endothelial cells
Myocytes
Osteoblast
Pericytes
Preadipocytes
Skeletal muscle
Smooth muscle
C
riskScore
CLP
CMP
Erythrocytes
GMP
HSC
Megakaryocytes
MEP
MPP
Platelets
D
riskScore
Astrocytes
Epithelial cells
Hepatocytes
Keratinocytes
Melanocytes
Neurons
Sebocytes
E
risk high low
value
*** ns ** *** ****
aDC
DC
iDC
Macrophages
Macrophages M1
F
risk high low
value
** *** * ** ns
Endothelial cells
Lymphatic Endothelial cells
Mesangial cells
Microvascular Endothelial cells
Myocytes

Supplementary Figure 4. Correlation between HPS and TME components validated by different algorithms.

(A-D) Correlation between HPS and the infiltration of different immune cell types in the TME, assessed using the xCell algorithm. Correlation coefficients are shown, with statistical significance indicated by asterisks (*, $p < 0.05$; **, $p < 0.01$; ***, $p < 0.001$).

(E-F) Infiltration of myeloid-derived immune cells and stromal cells in high- and low-risk groups, analyzed by the t-test. Statistical significance is indicated by asterisks (*, $p < 0.05$; **, $p < 0.01$; ***, $p < 0.001$; ****, $p < 0.0001$; ns, not significant).

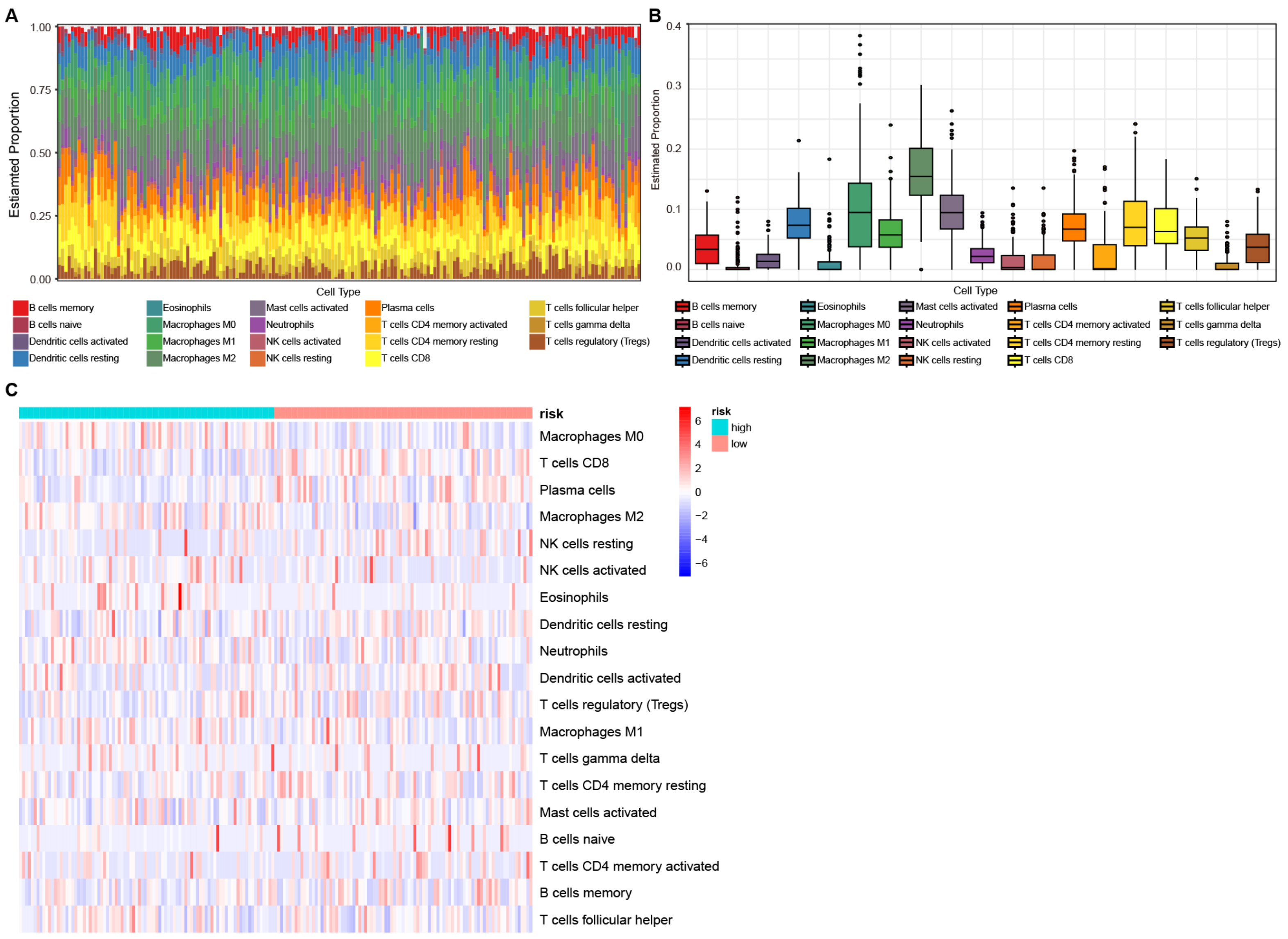

Supplementary Figure 5. Immune cell infiltration in the CRC tumor microenvironment (TME) and differential infiltration between high- and low-risk groups.

(A) Overall immune cell infiltration in CRC TME, presented as a stacked bar plot with immune cell proportions across samples, estimated using CIBERSORT.

(B) Individual immune cell infiltration in CRC TME, visualized using a boxplot.

(C) Differential immune cell infiltration between high- and low-risk groups, visualized using a heatmap.
