# Supplementary figures and images for: Hypoxia reconstructed colorectal tumor microenvironment weakening anti-tumor immunity: construction of a new prognosis predicting model through transcriptome analysis
Source: Front Immunol. 2024 Dec 6;15:1425687. doi: 10.3389/fimmu.2024.1425687 (PMC11659140; doi:10.3389/fimmu.2024.1425687)

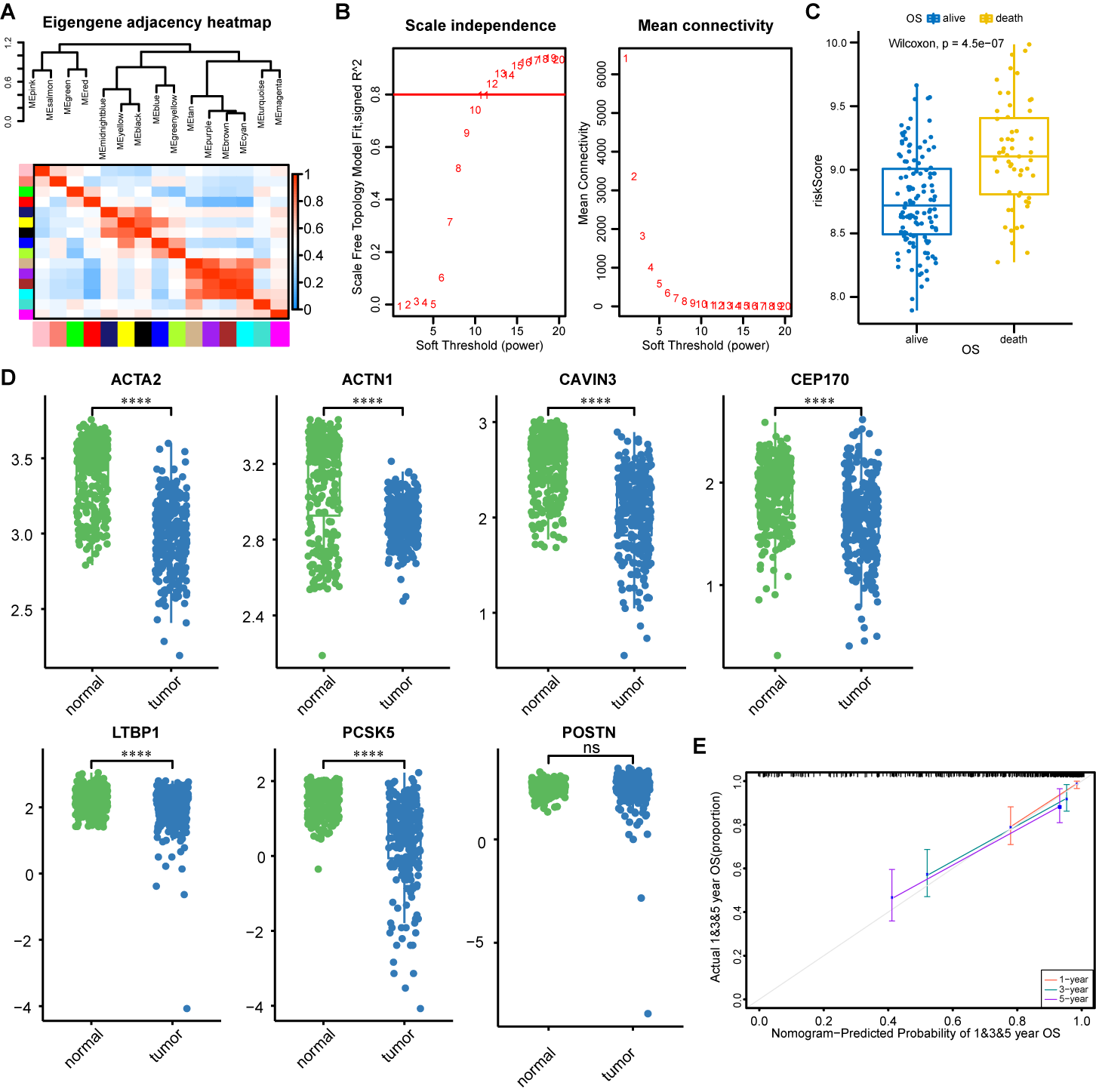

Supplement: Supplementary file 1 [file DataSheet1.zip › Supplementary_Material/Supplementary Figure 1.tif]

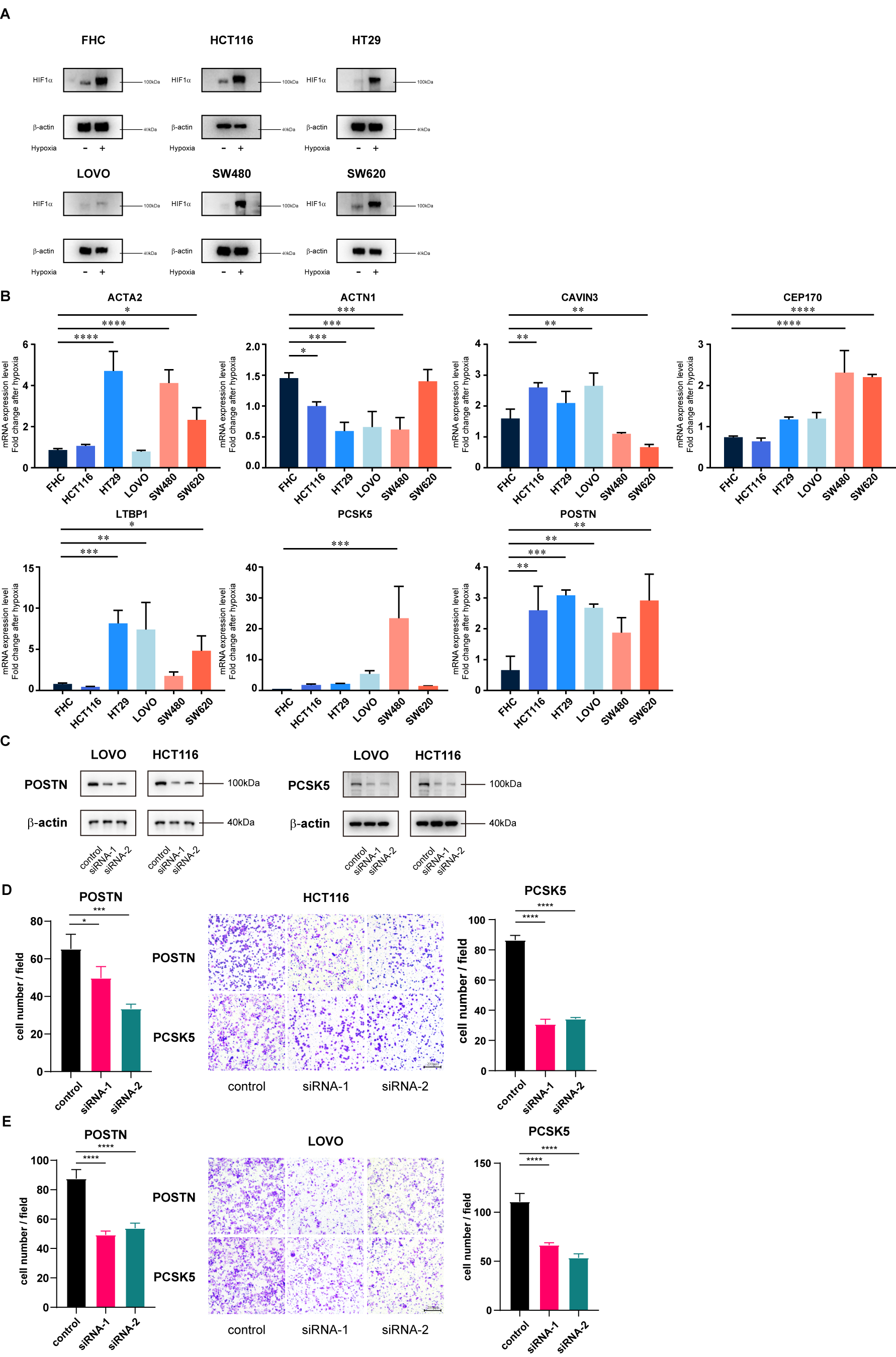

Supplement: Supplementary file 1 [file DataSheet1.zip › Supplementary_Material/Supplementary Figure 2.tif]

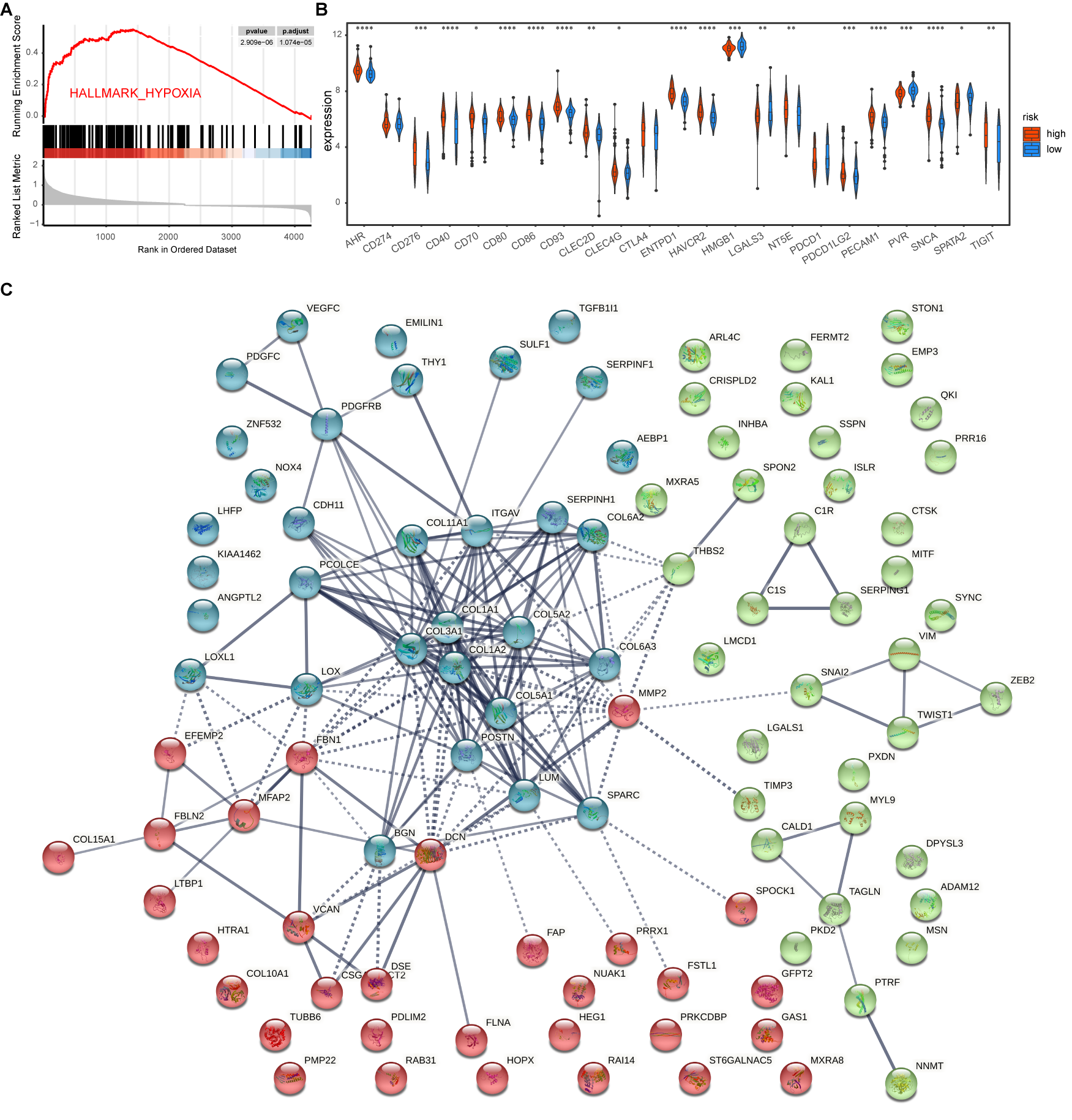

Supplement: Supplementary file 1 [file DataSheet1.zip › Supplementary_Material/Supplementary Figure 3.tif]

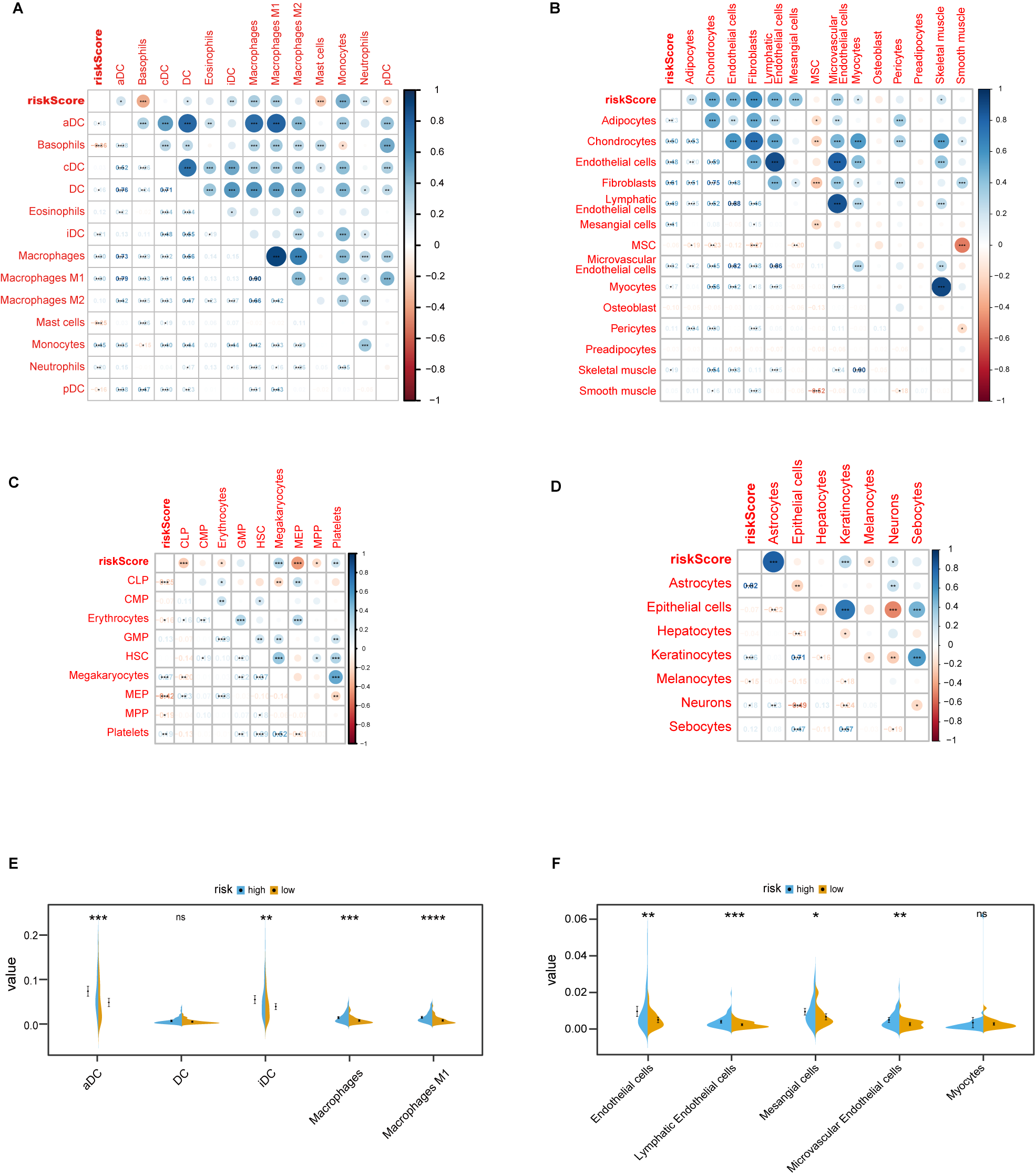

Supplement: Supplementary file 1 [file DataSheet1.zip › Supplementary_Material/Supplementary Figure 4.tif]

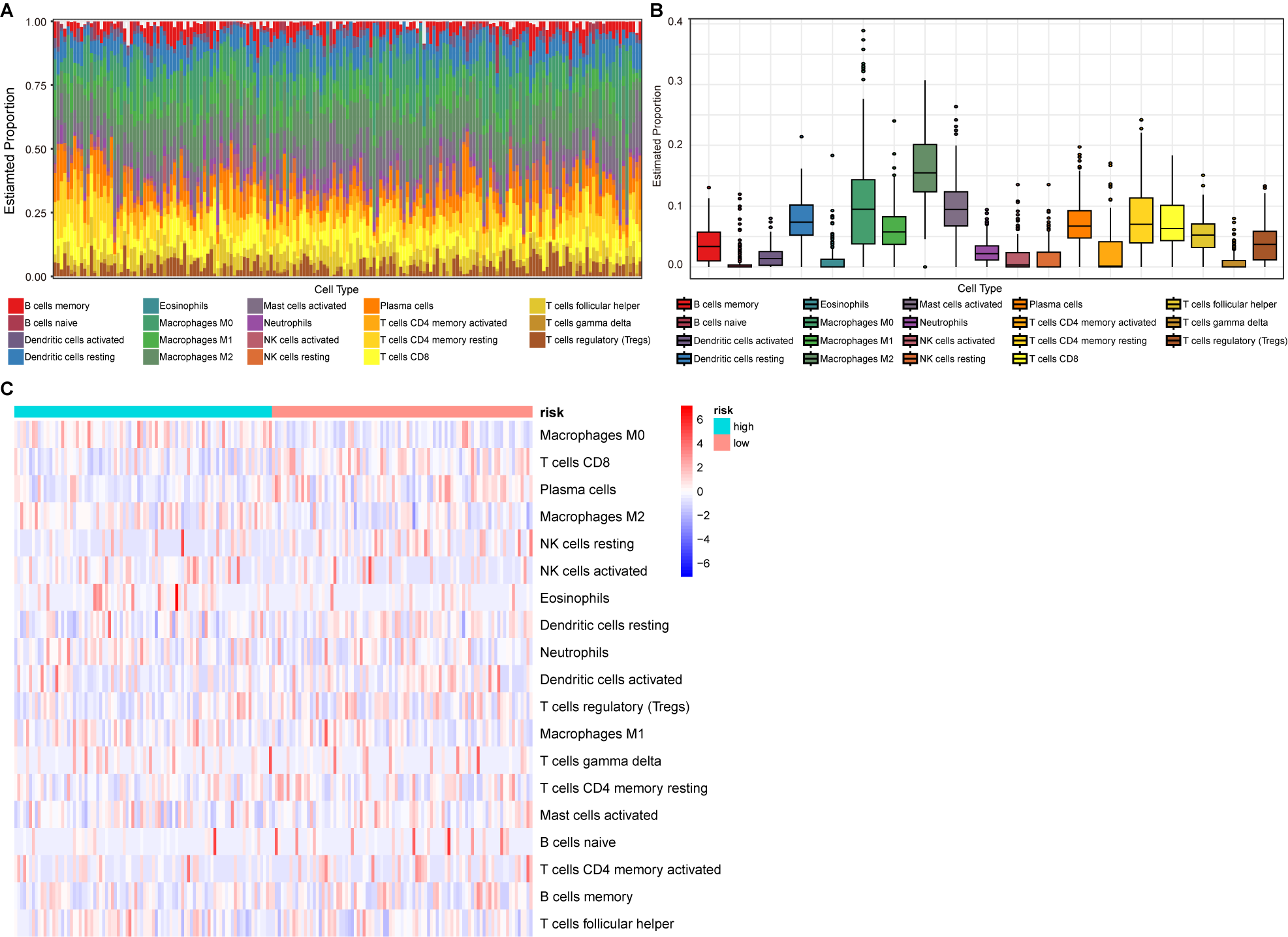

Supplement: Supplementary file 1 [file DataSheet1.zip › Supplementary_Material/Supplementary Figure 5.tif]
